# Senolytic-loaded asymmetric wound dressing for targeted senescent cell clearance in diabetic wound healing

**DOI:** 10.1016/j.mtbio.2025.102741

**Published:** 2025-12-24

**Authors:** Ming Zhang, Yamei Wang, Yao Dai, Yan Hu, Wanting Fu, Yuhao Zhao, Hanyu Ma, Di Zhang, Ying Chen, Yixuan Zhou, Lei Du, Jing Chang, Fang Liu, Shuyan Chen, Fei Wang, Dongdong Xiao, Zhen Li

**Affiliations:** a Department of Geriatrics, Xinhua Hospital, Shanghai Jiao Tong University School of Medicine, Shanghai, China; b Faculty of Flavour Fragrance and Cosmetics, Shanghai Institute of Technology, Shanghai, China; c Department of Pharmacy, Xinhua Hospital, Shanghai Jiao Tong University School of Medicine, Shanghai, China; d Department of Cardiovascular Surgery, Shanghai General Hospital, Shanghai, China; e Department of Urology, Ren Ji Hospital, Shanghai Jiao Tong University School of Medicine, Shanghai, China

**Keywords:** Diabetic foot ulcers, Chronic wound healing, Cellular senescence, Senolytics, ABT-263, Asymmetric wound dressing

## Abstract

Chronic wounds, especially diabetic foot ulcers, pose a major clinical challenge due to persistent inflammation, impaired angiogenesis, and cellular senescence. Senolytic therapies, which selectively eliminate senescent cells, have shown promise in promoting healing, but systemic toxicity limits their application. To address this, we developed an asymmetric fabric-based composite platform for localized senolytic delivery. Using single-cell RNA sequencing, we characterized senescence-associated alterations in human diabetic foot ulcer tissues and screened several senolytic candidates in skin fibroblasts and endothelial cells. Among these, navitoclax (ABT-263) emerged as the most effective senolytic. Incorporating ABT-263 into the fabric-based platform to prepare ABT-263-CGH, we confirmed its ability to reduce senescent cell burden while maintaining biocompatibility. In a diabetic mouse model, the ABT-263-CGH significantly enhanced wound healing, reduced senescence markers, and exhibited no detectable systemic toxicity. These findings highlight the potential of localized senolytic therapy using an asymmetric fabric-based wound dressing as a novel strategy for enhancing chronic wound healing, offering a promising therapeutic avenue for diabetic wound management, and paving the way for future clinical applications.

## Graphical abstract

**Figure:**
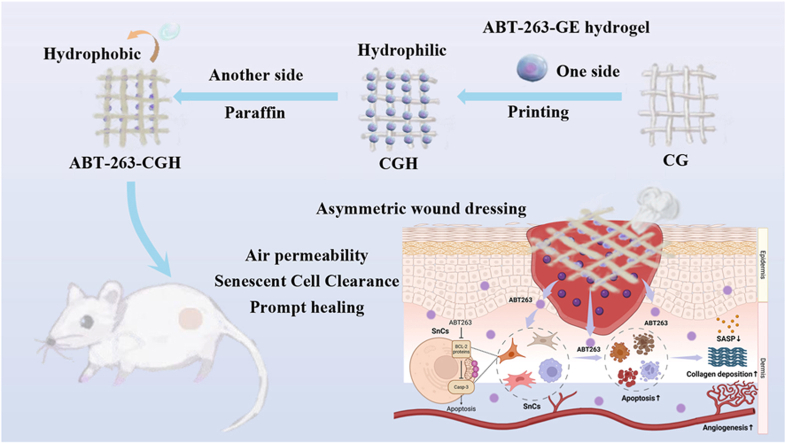

## 1 Introduction

Chronic wounds, particularly diabetic foot ulcers (DFUs), represent a significant and growing clinical challenge, especially among patients with diabetes. The impaired wound healing observed in these patients is attributed to a complex interplay of pathological factors, including persistent inflammation, dysregulated angiogenesis, and cellular senescence, which collectively hinder effective tissue repair [1]. Conventional wound management strategies, such as wound dressings and topical agents, primarily focus on symptom relief and infection control but often fail to promote complete wound closure, underscoring the urgent need for advanced therapeutic interventions that address the underlying cellular and molecular mechanisms of chronic wound pathology [2].

Recent studies have highlighted the critical role of cellular senescence in the pathophysiology of chronic wounds [3]. Senescent cells (SnCs), characterized by permanent cell cycle arrest, accumulate within chronic wound tissues, where they exert detrimental effects by adopting a senescence-associated secretory phenotype (SASP). This phenotype is marked by the excessive secretion of pro-inflammatory cytokines (e.g., IL-6, IL-1B), matrix metalloproteinases, and growth factors, which exacerbate local inflammation, impair extracellular matrix remodeling, and disrupt normal wound healing processes [4]. Furthermore, the persistence of SnCs in chronic wounds contributes to sustained oxidative stress and immune dysregulation, further delaying tissue regeneration [5]. Given these detrimental effects, targeted therapeutic strategies aimed at selectively eliminating SnCs have gained considerable interest in the field of regenerative medicine and wound care.

Senolytic agents, a class of drugs designed to induce apoptosis in SnCs selectively, have emerged as a promising therapeutic approach for improving chronic wound healing [6]. Among them, ABT-263, a Bcl-2 family inhibitor, has demonstrated potent senolytic activity by effectively clearing SnCs and attenuating SASP-related inflammatory responses [7]. However, the systemic administration of ABT-263 has been associated with dose-limiting toxicities, particularly thrombocytopenia, which significantly constrains its clinical application [8]. Consequently, localized delivery systems represent a more advantageous alternative, enabling targeted therapeutic action while effectively minimizing systemic adverse effects [9].

Biocompatible hydrogels have garnered increasing attention as versatile drug delivery platforms in wound care due to their ability to maintain a moist healing environment, promote cellular proliferation, and enable the controlled release of therapeutic agents [10]. In hydrogel-based drug-loaded wound dressings, commonly utilized therapeutic agents include peptides [11], nucleic acids [12], exosomes [13], and plant-derived bioactive compounds [14], among others. Some hydrogel dressings have poor breathability, which can increase the risk of wound infection and delay healing. Fabric-based dressings offer a promising approach to address this issue. Integrating functional hydrogel with gauze (GZ) creates a composite with enhanced moisture absorption to achieve optimal humidity control and air permeability [15]. Therefore, we propose an asymmetric composite dressing based on carboxymethylated gauze (CG), with a hydrophilic hydrogel layer for encapsulating and sustainably releasing the hydrophobic senolytic agent ABT263, and a hydrophobic layer to prevent external contamination. Incorporating the senolytic agents into the asymmetric composite dressing can localize the release of senolytics directly to the wound bed. This targeted delivery system holds the potential to enhance SnCs clearance, expedite wound healing, and mitigate the systemic toxicity concerns associated with oral senolytic drug administration.

**Scheme 1:**
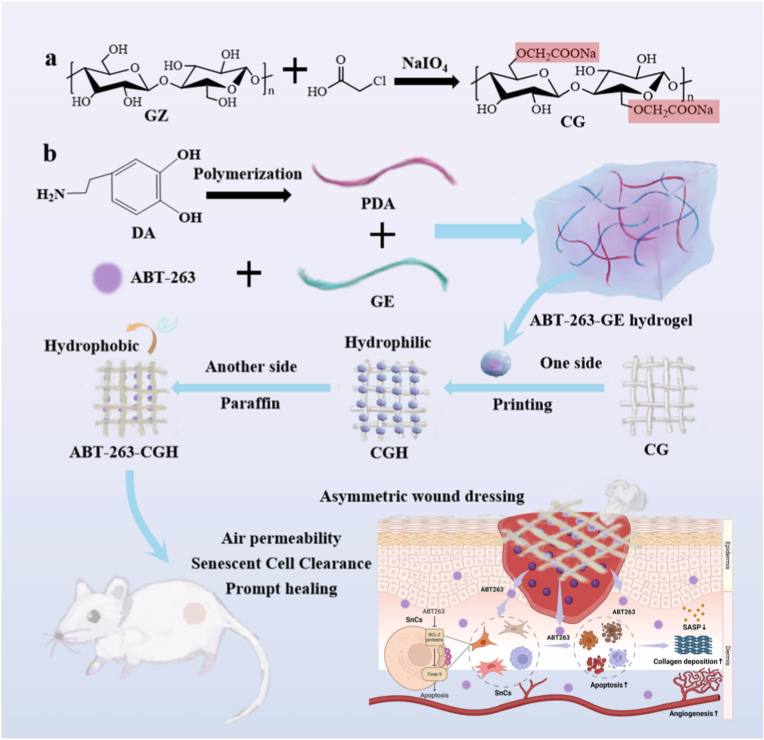
The diagram of the preparation process of ABT-263-loaded asymmetric wound dressing and its application in diabetic wound healing, and for targeted senescent cell clearance.

This study investigates the potential of an asymmetric fabric-based platform as a localized delivery system for the senolytic agent ABT-263 in chronic wound healing. To characterize cellular senescence in diabetic wounds, we examined skin tissues from patients with DFU and healthy individuals using single-cell transcriptomic analysis, senescence-associated β-galactosidase (SA-β-Gal) staining, as well as immunochemical detection of key senescence markers. To identify an optimal senolytic candidate, we evaluated the cytotoxicity and senescence-clearing effects of ABT-263, Dasatinib, Fisetin, Quercetin, and A1155463 in skin fibroblast and endothelial cell lines. The selected senolytic candidate was used to prepare an asymmetric wound dressing, and the wound healing performance was evaluated through a streptozotocin (STZ)-induced diabetic mouse model. This study highlights the promise of integrating senolytic therapy with biomaterial-based delivery platforms as a novel approach for accelerating wound repair in diabetic patients and lays the groundwork for future clinical applications (see Scheme 1).

## 2 Materials and methods

### 2.1 Materials

GZ was purchased from Hainuo Group Co., Ltd. (Shandong, China). The sodium hydroxide (NaOH), Monochloroacetic acid (ClCH_2_COOH), hydrochloric acid (HCl), gelatin (GE), ethanol, and dopamine (DA) were obtained from Sinopharm Chemical Reagent Co., Ltd (Shanghai, China). The ABT-263, diphenyl-1-picrylhydrazyl (DPPH), paraffin, and hexane were purchased from Shanghai Aladdin Bio-Chem Technology Co., Ltd. Deionized water (DI) was used in this study. Unless otherwise specified, all chemicals were purchased from commercial suppliers and used without further purification. DMEM (4.5 g/L glucose), penicillin-streptomycin, 0.25 % trypsin-EDTA, and fetal bovine serum were obtained from Gibco (Grand Island, NY, USA).

### 2.2 Human wound samples

This study included wound tissue samples from five DFU patients and five healthy people. All samples were collected following informed consent. Clinical characteristics were assessed for all participants. The results were summarized as mean ± standard deviation (SD) and evaluated using p-values for group comparison, as shown in Table S1. The study was approved by the Ethics Committee of Xinhua Hospital, Shanghai Jiao Tong University School of Medicine, China (Approval Number: XHEC-C-2024-145-1).

### 2.3 Diabetes mellitus mouse model

All animal protocols were approved by the Ethics Committee of Xinhua Hospital, Shanghai Jiao Tong University, and the Department of Model Animal Research at Xinhua Hospital (Approval Number: XHEC-F-2024-020). The mice were maintained on a standard chow diet under a 12-h light/12-h dark cycle with free access to food and water. C57BL/6J mice were used for the experiments. At 10 weeks of age, a single intraperitoneal injection of streptozotocin (STZ, HY-13753, USA) at 200 mg/kg, dissolved in citrate buffer (HY-B1610L, MCE, USA), was administered. On the ninth day, blood samples were collected from the tail to measure blood glucose levels, and a glucose concentration of ≥16.67 mmol/L was used to confirm the establishment of the diabetic model. Age-matched healthy mice were used as controls.

### 2.4 Histological analysis

For Hematoxylin and Eosin (H&E) staining, skin tissue sections were fixed in 4 % paraformaldehyde (G1101, Servicebio, SH, CHN), embedded in paraffin, and cut into sections. The sections were then deparaffinized, rehydrated, stained with hematoxylin for nuclear visualization, and counterstained with eosin to highlight cytoplasmic structures. For Masson's trichrome staining, the paraffin-embedded skin sections were deparaffinized and rehydrated, followed by staining with Weigert's iron hematoxylin to visualize nuclei. The sections were then incubated with a phosphomolybdic acid solution and stained with a mixture of acid fuchsin and aniline blue to differentiate collagen fibers from muscle and cytoplasmic components. Collagen fibers appeared blue, while muscle fibers and cytoplasm were stained red.

### 2.5 Senescence-associated β-galactosidase (SA-β-gal) staining

For skin samples, freshly harvested tissues were embedded in OCT compound, rapidly frozen, and sectioned using a cryostat. The cryosections were then air-dried. Both tissue sections and cultured cells were fixed and incubated overnight at 37 °C with SA-β-Gal staining solution (9860S, Cell Signaling, BSN, USA). After staining, samples were rinsed with phosphate-buffered saline (PBS, BL302A, Biosharp, GZ, CHN) and examined under a light microscope to assess cellular senescence. SA-β-Gal-stained tissue sections and cultured cells were imaged under a bright-field microscope using consistent exposure settings. For each group, fields were randomly selected from five different samples, and for each sample, the average of quantifications from five non-overlapping fields was used as the final statistical result. In tissue sections, the SA-β-Gal-positive area was identified using ImageJ through color thresholding, and the positive area ratio was calculated as (positive stained area/total analysis area) × 100 %. In cultured cells, the numbers of SA-β-Gal-positive and total cells were manually counted, and the percentage of positive cells was calculated as (positive cell count/total cell count) × 100 %. These metrics were used to assess the extent of cellular senescence in both sample types.

### 2.6 Immunofluorescent (IF) staining

Tissue sections were fixed, followed by evaporation and washing in PBS on a shaker. Antigen retrieval was performed, followed by an additional wash of the slides. Sections were blocked with 3 % bovine serum albumin (BSA, 36101 ES, Yeasen, CHN) at room temperature, then incubated overnight at 4 °C with anti-γ-H2A (ab177308, 1:500, Abcam, UK). After washing, secondary antibodies were applied in the dark at room temperature. The slides were then stained with DAPI (40728ES03, Yeasen, CHN) for 10 min, washed, dried, and mounted with anti-fade medium. Fluorescence intensity of cells was detected using an Olympus fluorescence microscope (BX51, Japan). IF stained samples were imaged using a fluorescence microscope under consistent exposure settings. For each group, fields were randomly selected from five different people/mice, and for each sample, the average of quantifications from five non-overlapping fields was used as the final statistical result. Positive cells exhibiting specific fluorescence signals were automatically counted using ImageJ software, while total cell numbers were determined by nuclear staining (DAPI). The percentage of positive cells was calculated as: (number of positive cells/total number of cells) × 100 %. This metric was used to evaluate the expression level of the target protein at the cellular level.

### 2.7 Immunohistochemical (IHC) staining

Tissue sections were deparaffinized in xylene, followed by rehydration through a graded ethanol series. Antigen retrieval was conducted by heating the slides in a citrate buffer (pH 6.0) using a microwave or pressure cooker. Endogenous peroxidase activity was blocked with hydrogen peroxide. Non-specific binding was minimized by incubating the slides with a blocking serum for 30 min at room temperature. Primary antibodies against p21 (ab109520, 1:250, Abcam, UK) were applied overnight at 4 °C. After washing, the slides were incubated with a secondary antibody conjugated to horseradish peroxidase for 30 min at room temperature. The antigen-antibody complex was visualized using DAB (K5007, DAKO, Denmark), followed by counterstaining with hematoxylin. The stained sections were dehydrated, cleared, and mounted for microscopic analysis. For each group, five different tissue samples were imaged under a bright-field microscope using identical settings. For each sample, the average of quantifications from five non-overlapping fields was used as the final statistical result. ImageJ software was used to isolate the positive staining and measure the integrated optical density (IOD) and stained area. The average optical density (AOD) was calculated as AOD = IOD/area to reflect protein expression levels.

### 2.8 Single-cell RNA-seq analysis

Single-cell transcriptomic analysis of human foot skin samples was performed using a publicly available dataset (GSE165816). Expression profiles from individual samples were merged and processed using the Seurat R package (v5.3). Cells were retained for downstream analyses if they expressed between 200 and 3500 genes, had total RNA counts between 500 and 20,000, and exhibited <15 % mitochondrial gene content. Gene expression data were normalized using the “LogNormalize” method with a scaling factor of 10,000. Highly variable genes were identified via the variance-stabilizing transformation (VST) method. Principal component analysis (PCA) was performed using the top 2000 variable genes, and the top 50 principal components were calculated. Batch effects were corrected using the Harmony algorithm, and the first 20 Harmony components were used to construct a shared nearest neighbor graph. Clustering was performed using the Louvain algorithm with a resolution parameter of 2. Clusters were manually annotated based on canonical marker genes. Dimensionality reduction and visualization were conducted using t-distributed stochastic neighbor embedding (t-SNE) based on the Harmony-corrected components. To assess cellular senescence, we computed a senescence module score for each cell using the SenMayo gene set. Cells with scores in the top 10 % were classified as senescent. Senescence scores were compared between the DFU and Healthy groups across different cell types, and log2 fold changes and false discovery rate (FDR)-adjusted p-values were calculated. Differential expression analysis was performed to generate a ranked gene list, which was then used for gene set enrichment analysis (GSEA) with the SenMayo gene set.

### 2.9 Preparation of the asymmetric dressing

For the carboxymethylation of original GZ, the mixture of ethanol and DI (V/V = 85/15) was used as solvent. Briefly, GZ (2 g) was immersed in NaOH solution (80 mL) at 10–20 °C for 30 min, and then ClCH_2_COOH solution (20 mL) was added. After being kept at 70 °C for 3 h, the pH of the system was adjusted to pH = 6–7 with HCl solution, and after that, the fabric was washed with the solvent. Finally, the fabric, called CG, was dried in an oven at 50 °C for 24 h. Here, the amounts of NaOH were 2 g, and the ClCH_2_COOH added were 0.5 g, 1 g, and 2 g, and the corresponding samples were named CG_0.5_, CG_1,_ and CG_2_.

To prepare the ABT-263-GE hydrogel, DA powder was dissolved in 100 mL DI, and the pH was adjusted to 7–8. After allowing DA to self-polymerize for 30 min under atmospheric conditions, the ABT-263 and 5 g GE were added and dissolved to form the ABT-263-GE hydrogel, and the concentration of ABT-263 was 10 μM. 5 g GE was dissolved directly in 100 mL DI, and the solution was cooled to obtain the GE hydrogel. The preparation of the asymmetric ABT-263-CGH included three steps. Firstly, GZ was carboxymethylated to form CG as the substrate; secondly, the ABT-263-GE hydrogel was printed onto one side of the CG using a screen-printing method with a mesh size of 0.6 mm to obtain ABT-263-GE hydrogel-loaded CG (CGH). Then, paraffin was deposited on the other side of the CG, specifically by spraying a 6.25 % paraffin solution onto the CG 20 times with a spray bottle and allowing it to air dry to create a hydrophobic surface.

### 2.10 Characterizations

#### 2.10.1 Basic characterizations

The infrared spectra and contact angles of GZ, CGs, and ABT-263-CGH were investigated by a Fourier transform infrared spectroscopy (FT-IR, Spectrum Two, China) and a Theta contact angle analyzer (Biolin, Sweden), respectively. A scanning electron microscope (SEM, TM3030, Japan) was used to obtain the morphology images of GZ, CGs, hydrogels, and ABT-263-CG. The universal material testing machine (UH6502) was used to test the mechanical properties of GZ and CGs, where the tensile rate was 20 mm/min. Besides, an optical microscope (OM, ECLIPSE E100, Japan), X-Ray diffractometer (XRD, D/max-2500PC, Japan), and moisture permeability tester (YG601H, China) were used in this study to characterize the properties of samples. To analysis the rheological property of hydrogels, the curves of the storage moduli (G′) and loss modulus (G″) was obtained using a HAAKE-MARS60 rheometer. The temperature was set at 20 °C, the diameter of parallel plate geometry was 25 mm, and the height of the sample was 2 mm. The G′ and G″ of hydrogels were measured with the frequencsy in the range of 0.1–5 Hz when the constant strain was set as 1 %.

#### 2.10.2 Water and blood absorption properties

For the GZ, CGs, and ABT-263-CGH, water absorption and blood absorption properties were investigated. The fabric was snipped into squares with sides of 1 cm and weighed (W_initial_), then immersed in DI or anticoagulated rat blood for 30 min. After that, it was taken out and hung in the air for 10 s, and the weight was recorded (W_wet_). Water retention rate or blood absorption rate (W_r_) of the hydrogel was calculated by the following equation (1).

(1) Wr=(Wwet‐Winitial)×100%Winitial

#### 2.10.3 Antioxidant property of GE and ABT-263-GE hydrogels

The antioxidant performance of the samples was evaluated by testing their efficiency in capturing DPPH free radicals. First, 1.9716 mg of DPPH powder was dissolved in 100 mL of anhydrous ethanol to prepare the DPPH solution. The hydrogel was then dispersed in the DPPH solution and incubated in the dark for 30 min. The DPPH solution served as the control group (A_DPPH_). After the reaction, the absorbance of the sample within the 350–700 nm range was measured using a UV–Vis spectrophotometer, and the absorbance at 517 nm was recorded (A_sample_). The antioxidant efficiency of the sample (E_sample_) was calculated using the following equation (2).

(2) Esample=(ADPPH‐Asample)×100%ADPPH

#### 2.10.4 Release performance and kinetic analysis of ABT-263-GE hydrogel

A certain amount of ABT-263 powder was weighed and dissolved in deionized water at concentrations of 0.04 mg/mL, 0.05 mg/mL, 0.06 mg/mL, 0.07 mg/mL, and 0.08 mg/mL. The absorbance values at the maximum absorption wavelength are measured using a UV–Vis Spectrophotometer (Hitachi High-Tech Corporation, Japan), and the calibration curve of ABT-263 was obtained. Then, ABT-263-GE hydrogel was soaked in deionized water, placed at 4 °C. The release rate of ABT-263 was calculated using the following equation (3).

(3) $$\text{Release}\;\text{rate}\;\text{of}\;\text{ABT}-263=\frac{M_{T}}{M_{\max}}\times 100\%$$

Where MT was the amount of ABT-263 released at time T, and Mmax was the initial amount of ABT-263.

The release kinetics of ABT-263 were analyzed using the Avrami equation expressed in linear form in equation (4).

(4) ln(−ln(1−Y))=lnk+nlnt

Where Y was the release rate of ABT-263, t was the release time, k was the release rate constant, and n was the Avrami parameter that characterizes the release mechanism.

### 2.11 Cellular senescence models

Cellular senescence was induced by co-cultivating L929 and HUVECs in a cell culture medium supplemented with etoposide (ETO, HY-13629, MCE, USA) at a concentration of 2.5 μM or Advanced glycation end products-modified bovine serum albumin (AGEs, BGT-CMP-100, Biogradetech) 200 μg/mL for a duration of 48 h.

### 2.12 Cell viability assay

L929 and HUVECs were seeded into 96-well plates at a density of 5 × 10^4^ cells/well in 100 μL of complete medium, with three replicates per group. During the drug screening phase, cells were divided into vehicle-treated versus senescence-induced groups. Two senescence models were employed: an ETO-induced model and an AGEs-induced model. In the ETO-induced model, senolytic candidates—ABT-263 (S1001, Selleck, USA), Fisetin (S2298, Selleck, USA), A1155463 (S7800, Selleck, USA), Dasatinib (S7782, Selleck, USA), Quercetin (S2391, Selleck, USA), and the combination of Dasatinib plus Quercetin (D + Q)—were tested. The AGEs-induced L929 cell model was also evaluated with ABT-263 and D + Q. For each tested compound, vehicle-treated versus senescent cells were exposed for 48 h to a range of concentrations (0.0625–128 μM for single agents; for D + Q, Dasatinib at 0.625–1280 nM combined with Quercetin at 0.0625–128 μM). Following treatment, 10 μL of CCK-8 reagent was added to each well and incubated at 37 °C for 30 min. The optical density (OD) at 450 nm was measured to determine the half-maximal inhibitory concentration (IC_50_). The selectivity index (SI) was calculated by dividing the IC_50_ value for proliferating (vehicle-treated) cells by that for senescent cells, reflecting the compound's ability to selectively target senescent cells while sparing healthy, proliferating cells.

During the material effectiveness validation phase, CGH and CGH loaded with 10 μM ABT-263 were sterilized using ^60^Co irradiation and incubated in a small volume of DMEM (12491015, Gibco, USA) supplemented with 10 % fetal bovine serum at 37 °C with continuous stirring for 48 h. The extract was collected by centrifugation at 100*g*, and the supernatant was used to culture senescent HUVECs and L929 cells, while the senescent model group received an equal volume of complete medium as a control. Cell viability was assessed by measuring the OD at 450 nm daily for seven consecutive days.

For material safety evaluation, proliferating HUVECs and L929 cells were treated with the extract from CGH, while vehicle-treated cells served as controls. OD values were measured on days 1, 3, 5, and 7 using the CCK-8 assay to evaluate cell viability.

### 2.13 Calcein/propidium iodide (PI) staining

HUVECs and L929 cells were treated with the extracted solution above or an equivalent volume of complete medium for 48 h. After treatment, cells were washed with PBS and incubated with 1 μmoL/ml PI and Calcein AM (C2015, Beyotime, CHN) in the dark at 37 °C for 30 min. Stained cells were then analyzed using a fluorescence microscope.

### 2.14 Protein extraction and western blotting

Cells were lysed in RIPA buffer (P0013B, Beyotime, CHN) supplemented with PMSF (ST506, Beyotime, CHN). The total protein concentration was determined using the BCA assay (P0011, Beyotime, CHN). Protein samples were separated by 12.5 % SDS-PAGE (PG004, Epizym, CHN) and transferred onto PVDF membranes (IPVH00010, Millipore, USA). The membranes were blocked with 5 % non-fat milk in TBST (ST671, Beyotime, CHN) for 2 h at room temperature, followed by incubation with primary antibodies against p21 (ab109520, 1:1000, Abcam, UK) and α-tubulin (AF2827, 1:2000, Beyotime, CHN) overnight at 4 °C. After washing with TBST, the membranes were incubated with HRP-conjugated secondary antibodies (HRP-labeled goat anti-mouse IgG, A0216, 1:1000, Beyotime, CHN, or HRP-labeled goat anti-rabbit IgG, A0208, 1:1000, Beyotime, CHN) for 1 h at room temperature. Protein bands were visualized using an enhanced chemiluminescence (ECL, WBKIS0100, Millipore, USA) detection system, and relative protein expression levels were quantified using ImageJ software (Bio-Rad, USA).

### 2.15 RNA extraction and quantitative real-time polymerase chain reaction (RT-qPCR)

Total RNA was extracted from cultured cells using RNA-easy isolation reagent (R701-01, Vazyme, CHN) according to the manufacturer's instructions. The RNA concentration and purity were assessed using a NanoDrop spectrophotometer (Thermo Fisher). Reverse transcription was performed using a cDNA synthesis kit (RR036A, Takara Bio, Japan). RT-PCR was conducted using a SYBR Green Master Mix (11201ES08, Yeasen, CHN) on the ABI QuantStudioTM3 system (CT, USA). The gene expression levels of *p16, p21, Il-1a, Il-1b, Cxcl1,* and *Mmp10* were analyzed, with *Gapdh* serving as the internal control. Table S2 lists the primers utilized in this investigation. The relative mRNA expression levels were calculated using the 2^−ΔΔCt^ method. All reactions were performed in triplicate to ensure reproducibility.

### 2.16 EdU proliferation assay

Cell proliferation was assessed using the BeyoClick™ EdU Cell Proliferation Kit with Alexa Fluor 594 (C0078S, Beyotime, Shanghai, China). L929 cells were incubated with EdU working solution at a final concentration of 10 μM for 2 h, respectively. After incubation, the culture medium was removed, and the cells were fixed with fixation solution at room temperature for 15 min. The cells were then washed with washing buffer and permeabilized with 0.3 % Triton X-100 at room temperature for another 15 min. Following another wash, a freshly prepared Click reaction solution was added, and the cells were incubated in the dark at room temperature for 30 min. After removing the Click solution and washing, Hoechst 33342 working solution was added and incubated at room temperature for 10 min, followed by a final wash. Fluorescence signals were then visualized using an Olympus fluorescence microscope.

### 2.17 Wound model

Twelve-week-old diabetic and non-diabetic mice of matched ages were anesthetized with a combination of 2 % isoflurane and oxygen at a flow rate of 0.2–0.3 L/min. A full-thickness circular punch wound (5 mm in diameter) was created on the dorsal skin of diabetic mice, and the wound was fixed using adhesive glue and a pad ring to establish a chronic skin wound model. The wound dressings were changed every other day. Wound healing was assessed by capturing images on days 0, 3, 7, 10, and 14, followed by measurement and analysis using ImageJ software. The wound area was normalized based on the captured images, and the percentage of wound closure was calculated using the formula: (wound area on day n/wound area on day 0) × 100 (%).

### 2.18 Liver and kidney function assessment

A full-thickness circular punch wound (5 mm in diameter) was created on the dorsal skin of twelve-week-old wild-type C57 mice, which were divided into two groups. One group was treated with CGH, while the other remained untreated as a control until complete wound healing. Blood samples were collected via orbital sampling, and liver and kidney tissues were harvested post-mortem. Serum levels of alanine aminotransferase (ALT), aspartate aminotransferase (AST), and creatinine (CREA) were measured, and liver and kidney tissues were subjected to H&E staining for histological analysis.

### 2.19 Skin hydration measurement

Prior to measurement, mice were acclimated for 30 min in a controlled environment (22 °C, 55 % humidity). Skin hydration was assessed using a Corneometer® CM825 probe (Courage + Khazaka electronic GmbH, Cologne, Germany) in accordance with the manufacturer's instructions. The probe was gently applied to the dorsal skin surface to record hydration levels. Three technical replicates were performed for each mouse.

### 2.20 Skin elasticity pinch test

Skin elasticity was evaluated by gently pinching the dorsal skin of each mouse between the thumb and forefinger and lifting it to the highest point possible without raising the mouse. The time required for the skin to return to its normal state after release was recorded in seconds. Three technical replicates were conducted for each mouse at each time point.

### 2.21 Statistical analysis

The data presented in this study are expressed as mean ± SEM from independent experiments. The differences were analyzed by Student's t-test, one- or two-way ANOVA. ∗P < 0.05, ∗∗P < 0.01, ∗∗∗P < 0.001 and ns, no significance.

## 3 Results

### 3.1 Senescent cells accumulate in chronic diabetic wounds

**Fig. 1:**
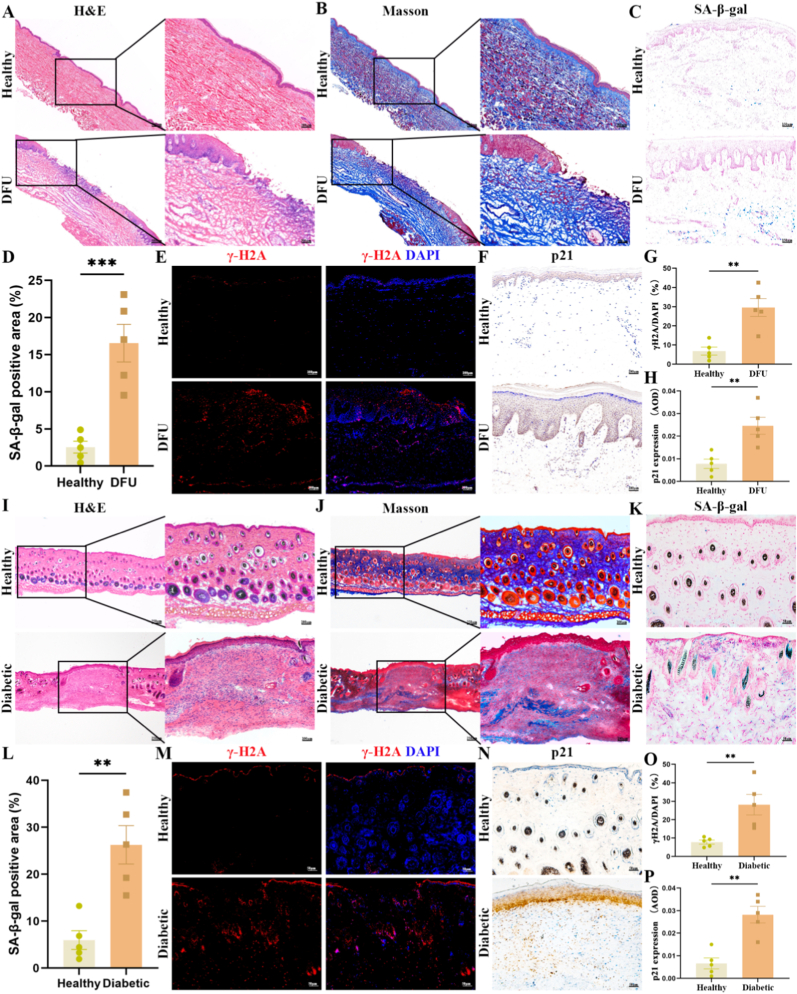
**Elevated cellular senescence markers in chronic diabetic wounds.** (A–B) Representative images of H&E (A) and Masson's trichrome (B) staining of skin tissues from healthy individuals (n = 5) and patients with diabetic foot ulcers (DFU, n = 5). (C) Representative images of SA-β-Gal staining of skin tissues in healthy individuals (n = 5) and DFU patients (n = 5). (D) Quantification of SA-β-Gal-positive cells is shown in (C). (E) Representative images of immunofluorescence staining of γ-H2AX in skin tissues from healthy individuals (n = 5) and DFU patients (n = 5). (F) Representative images of immunohistochemical staining of p21 from healthy individuals (n = 5) and DFU patients (n = 5). (G–H) Quantification of γ-H2AX-positive nuclei (G) and p21-positive staining (H, AOD method) from images shown in (E) and (F), respectively. (I–J) Representative images of H&E (I) and Masson's trichrome (J) staining of skin tissues from healthy controls (n = 5) and diabetic wound model mice (n = 5). (K) Representative images of SA-β-Gal staining of skin tissues in healthy controls (n = 5) and diabetic wound model mice (n = 5). (L) Quantification of SA-β-Gal-positive cells is shown in (K). (M) Representative images of immunofluorescence staining of γ-H2AX in skin tissues from healthy controls (n = 5) and diabetic wound model mice (n = 5). (N) Representative images of immunohistochemical staining of p21 from healthy controls (n = 5) and diabetic wound model mice (n = 5). (O–P) Quantification of γ-H2AX-positive nuclei (O) and p21-positive staining (P, AOD method) shown in (M) and (N), respectively. Values represent mean ± SEM. Statistical analysis was performed using Student's t-test and/or one-/two-way ANOVA. ∗P < 0.05, ∗∗P < 0.01, ∗∗∗P < 0.001.

To investigate the presence of cellular senescence in chronic diabetic wounds, we first examined skin samples from the wound sites of DFU patients and healthy controls. Hematoxylin-eosin (H&E) and Masson's trichrome staining revealed pronounced hyperkeratosis, epidermal thickening, disorganized and fibrotic collagen deposition in the dermis, and inflammatory cell infiltration in DFU tissues (Fig. 1A and B). SA-β-Gal staining showed marked positivity in the dermis of diabetic wounds (Fig. 1C and D). Full-thickness skin immunofluorescence revealed upregulation of γ-H2AX, a marker of DNA damage and cellular aging (Fig. 1E and G). At the same time, immunohistochemistry showed elevated expression of p21 and p16 in the skin, with signals concentrated at the epidermal–dermal junction (Fig. 1F and H, and S1A). Moreover, Western blot analysis revealed a significant upregulation of p21 expression in the skin tissue of the DFU group (Fig. S1B). These findings indicate that cellular senescence is significantly elevated in diabetic wound tissue compared to normal skin.

To corroborate these observations in vivo, we established a diabetes mellitus mouse model via a single high-dose intraperitoneal injection of STZ. H&E and Masson staining revealed mild but increased scattered inflammatory cell infiltration, accompanied by active epithelialization (Fig. 1I and J). The senescence markers in murine wounds recapitulated the patterns observed in human samples, with elevated SA-β-Gal activity (Fig. 1K and L). Immunofluorescence similarly showed enhanced γ-H2AX expression (Fig. 1M and O), and immunohistochemistry confirmed increased levels of p21 and p16 (Fig. 1N and P, and S1C). Consistently, Western blot analysis demonstrated a marked upregulation of p21 expression (Fig. S1D). Collectively, these human and animal data provide compelling evidence implicating cellular senescence in the pathogenesis of chronic diabetic wounds.

### 3.2 Single-cell transcriptomic analysis reveals multilineage senescence in chronic diabetic wounds

**Fig. 2:**
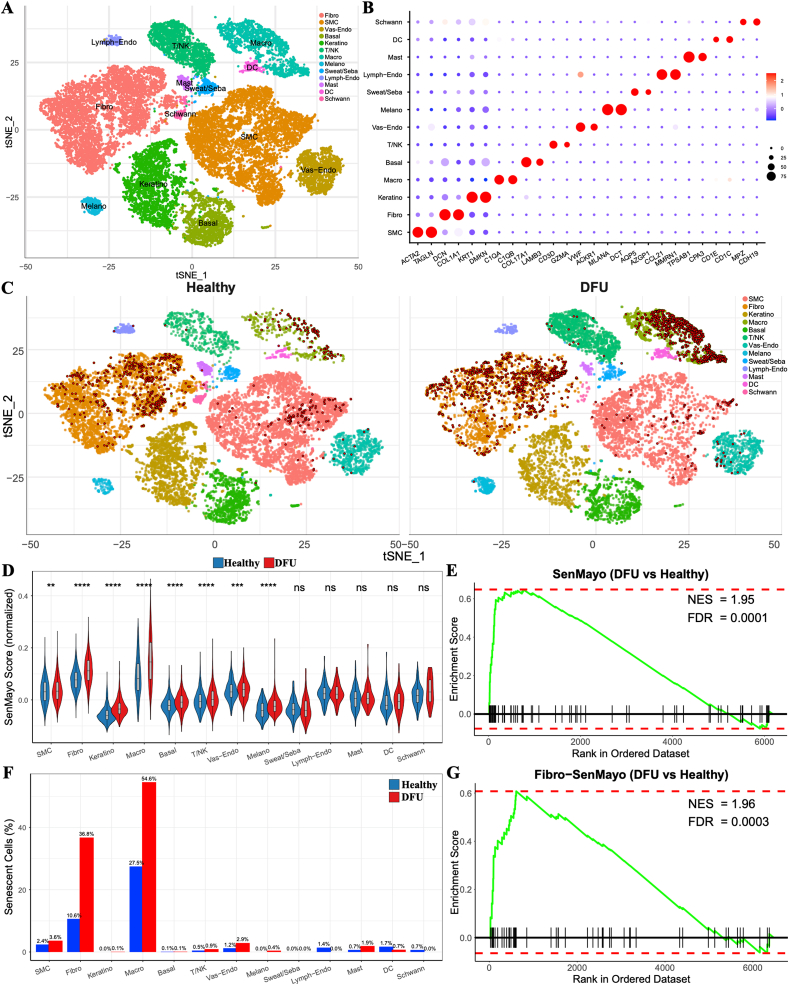
**Single-cell transcriptomic profiling reveals accumulation and heterogeneity of senescent cells (SnCs) in diabetic foot ulcer (DFU) skin.** (A) t-SNE plot showing clustering of 24,691 cells from healthy (n = 3) and DFU (n = 3) foot skin into 13 cell types based on canonical markers. (B) Dot plot of representative marker genes per cell type. Dot size: % of expressing cells; color: average expression. (C) t-SNE plot highlighting SnCs (red; top 10 % SenMayo score), predominantly enriched in DFU samples. (D) Comparison of SenMayo scores across cell types; eight types show significantly higher scores in DFU. (E) GSEA showing significant enrichment of SenMayo genes in DFU skin (NES = 1.95, FDR <0.01). (F) Proportion of SnCs across cell types in DFU, highlighting fibroblasts as a major senescent population (36.8 %). (G) GSEA plot showing significant enrichment of SenMayo genes in DFU-derived fibroblasts (NES = 1.96, FDR <0.01), suggesting their central role in wound senescence. (For interpretation of the references to color in this figure legend, the reader is referred to the Web version of this article.)

To identify the senescence of various cell types in chronic diabetic wounds, we analyzed single-cell RNA sequencing (scRNA-seq) data derived from foot skin tissues of healthy individuals (n = 3) and DFU patients (n = 3). A total of 24,691 cells (13,664 from healthy samples and 11,027 from DFU samples) were classified into 13 distinct cell types based on dimensionality reduction analysis and established cell-specific marker genes (Fig. 2A, Fig. S2A). These included smooth muscle cells (SMC: ACTA2+, TAGLN+), fibroblasts (Fibro: DCN+, COL1A1+), keratinocytes (Keratino: KRT1+, DMKN+), macrophages (Macro: C1QA+, C1QB+), basal cells (Basal: COL17A1+, LAMB3+), T and natural killer cells (T/NK: CD3D+, GZMA+), vascular endothelial cells (Vas-Endo: VWF+, ACKR1+), melanocytes (Melano: MLANA+, DCT+), sweat and sebaceous gland cells (Sweat/Seba: AQP5+, AZGP1+), lymphatic endothelial cells (Lymph-Endo: CCL21+, MMRN1+), mast cells (Mast: TPSAB1+, CPA3+), dendritic/Langerhans cells (DC: CD1E+, CD1C+), and Schwann cells (Schwann: MPZ+, CDH19+) (Fig. 2B, Fig. S2B).

Given the heterogeneity of SnCs and the limited detectability of canonical senescence markers such as p16^INK4a^ in whole-transcriptome analyses, we employed the SenMayo gene set (125 genes) to identify SnCs at the single-cell level. As shown in Fig. 2C and D, DFU samples exhibited a marked increase in SnCs (defined as cells with SenMayo scores in the top 10 %) compared to healthy controls. Consistently, gene set enrichment analysis (GSEA) revealed significant enrichment of SenMayo genes in DFU samples (NES = 1.95, FDR <0.01) (Fig. 2E), further confirming the accumulation of SnCs in DFU skin. Moreover, comparison of SenMayo scores across cell types between groups indicated significantly elevated senescence signatures (p < 0.01) in eight cell populations in DFU: SMCs, Fibroblasts, Keratinocytes, Macrophages, Basal cells, T/NK cells, Vascular endothelial cells, and Melanocytes (Fig. 2D, Fig. S2C). Cell-type-specific analysis of senescent cell proportions showed that (Fig. 2F), apart from macrophages, known for their inflammatory roles, senescent fibroblasts accounted for the highest proportion of SnCs in DFU (36.8 %). GSEA analysis of fibroblasts also demonstrated significant SenMayo gene enrichment in DFU fibroblasts (NES = 1.96, FDR <0.01) (Fig. 2G), suggesting that fibroblasts may represent the predominant senescent cell population in DFU lesions.

In addition, CDKN1A (p21), as well as anti-apoptotic members of the Bcl-2 family (BCL2, BCL2L1, BCL2L2, and MCL1) were significantly increased in cell subpopulations with high SenMayo scores, particularly within fibroblasts (Fig. S3A and S3B). To better characterize the heterogeneity and senescence-associated roles of fibroblasts in chronic wounds, we performed subclustering analysis to identify distinct fibroblast subtypes. As shown in Fig. S3C, four fibroblast subpopulations were identified: ECM-producing, pro-inflammatory, pro-regenerative, and stress-adaptive. These classifications were based on marker gene expression and GO enrichment analysis (Fig. S3D–S3H). Notably, both SenMayo scores and p21 expression were markedly elevated in the pro-inflammatory fibroblast subset (Fig. S3I and S3J), suggesting that this subtype may constitute a key senescent cell population contributing to impaired healing in chronic diabetic wounds.

### 3.3 Identification of ABT-263 as an effective senolytic in chronic diabetic wounds via screening

**Fig. 3:**
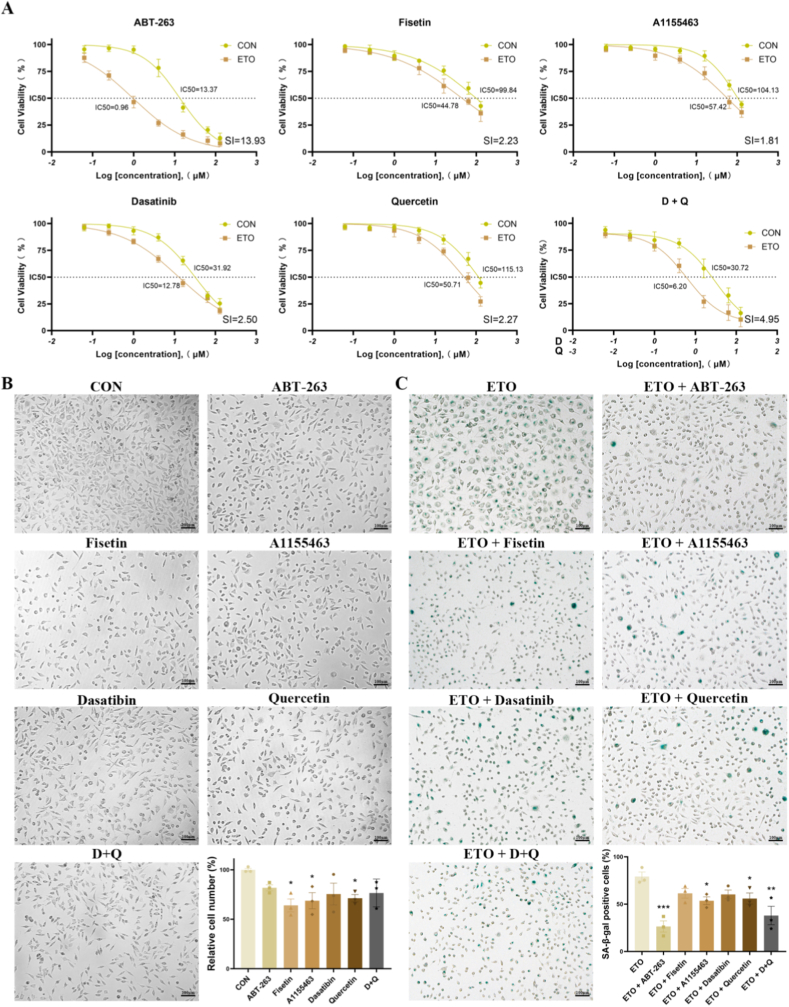
**Screening and evaluation of senolytic agents in vehicle-treated and etoposide (ETO)-treated L929 cells.** (A) Cell viability assays were performed on vehicle-treated (CON, n = 3) and ETO-treated (n = 3) L929 cells exposed to varying concentrations of ABT-263, Fisetin, A1155463, Dasatinib, Quercetin, and D + Q for 48 h. Cell viability was assessed using CCK-8 and expressed as a percentage relative to untreated controls. Dose-response curves and IC_50_ values were calculated for each compound. The selectivity index (SI) was determined by dividing the IC_50_ in proliferating cells by that in senescent cells. (B) Cytotoxicity of each senolytic agent (n = 3 for each agent), shown with representative images, at its optimal concentration after 48-h treatment of vehicle-treated L929 cells, assessed via cell count quantification. (C) Representative images of SA-β-Gal staining of ETO-treated senescent L929 cells treated with each senolytic agent (n = 3 for each agent) at its optimal concentration for 48 h, with quantification of SA-β-Gal-positive cell percentages. Values represent mean ± SEM. Statistical analysis was performed using Student's t-test or one-/two-way ANOVA. ∗P < 0.05, ∗∗P < 0.01, ∗∗∗P < 0.001.

In light of the above findings and previous studies [[16], [17], [18]], which have confirmed the critical role of SnCs in the development and progression of chronic diabetic wounds, targeting SnCs for elimination represents a highly promising therapeutic strategy for enhancing wound healing. To identify the most suitable senolytic agent for treating chronic wounds, we evaluated the effects of five individual compounds with known senolytic activity, each capable of inducing apoptosis in SnCs via distinct mechanisms: ABT-263 (a BCL-2/BCL-XL inhibitor), Fisetin (a PI3K/AKT pathway inhibitor), A1155463 (a selective BCL-XL inhibitor), Dasatinib (a Src family kinase inhibitor), and Quercetin (a flavonoid that inhibits PI3K and other pro-survival pathways. In addition, the senolytic cocktail Dasatinib and Quercetin (D + Q) was also included. Considering that single-cell transcriptomic analyses have suggested fibroblasts represent the predominant senescent cell population in DFU lesions and play a key role in extracellular matrix remodeling, we first evaluated the senolytic effects of candidate drugs on senescent murine skin fibroblasts (L929) in vitro. Cellular senescence in ETO-treated cells was validated by SA-β-gal staining (Fig. S4A), elevated expression of p16 and p21 detected by qPCR (Fig. S4B), and reduced EdU incorporation (Fig. S4C), confirming effective senescence induction. Following 48-h treatment with various concentrations of senolytics, cell viability was assessed in both vehicle-treated and ETO-treated cells. The appropriate concentration range for each compound was identified as the range in which the viability of ETO-treated (senescent) cells dropped below 50 %, while that of vehicle-treated (proliferating) cells remained above 50 %, thereby ensuring selective clearance of senescent cells (SnCs) while preserving healthy cells. The optimal dose for each agent was defined as the half-maximal inhibitory concentration (IC_50_) for SnCs and was used for subsequent experiments. The selectivity index (SI) was calculated by dividing the IC_50_ value in proliferating cells by that in senescent cells. Among all tested compounds, ABT-263 exhibited the highest SI (Fig. 3A). At their respective optimal concentrations, ABT-263 demonstrated the lowest cytotoxicity to vehicle-treated L929 cells, compared to the other senolytic regimens (Fig. 3B). Subsequently, senescent L929 cells were treated with the optimal concentrations of each compound for 48 h. SA-β-gal staining revealed that ABT-263 achieved the most efficient SnC clearance (Fig. 3C). To further validate our screening results, we established an AGEs-induced senescence model (Fig. S5A–5C). Similar to the ETO-induced model, ABT-263 more effectively reduced senescent fibroblasts in this model (Fig. S5D).

Given the critical role of endothelial cells in the angiogenic phase of wound healing, and the significant accumulation and enrichment of SenMayo genes (NES = 1.45, FDR <0.05) in DFU lesions as revealed by single-cell transcriptomic analysis (Fig. S2C and S2D), we also selected human umbilical vein endothelial cells (HUVECs) for in vitro drug screening. Using the same approach as with fibroblasts, we assessed the viability of both vehicle-treated and ETO-treated HUVECs treated with senolytic agents across a range of concentrations to determine the optimal dose for each drug (Fig. S6A). Notably, ABT263 exhibited the lowest cytotoxicity to vehicle-treated HUVECs (Fig. S6B) while achieving the most effective clearance of SnCs at its optimal concentration (Fig. S6C). Based on these findings, ABT-263 was selected as the optimal senolytic candidate for promoting the healing of chronic diabetic wounds.

### 3.4 Establishment and properties of asymmetric wound dressing

**Fig. 4:**
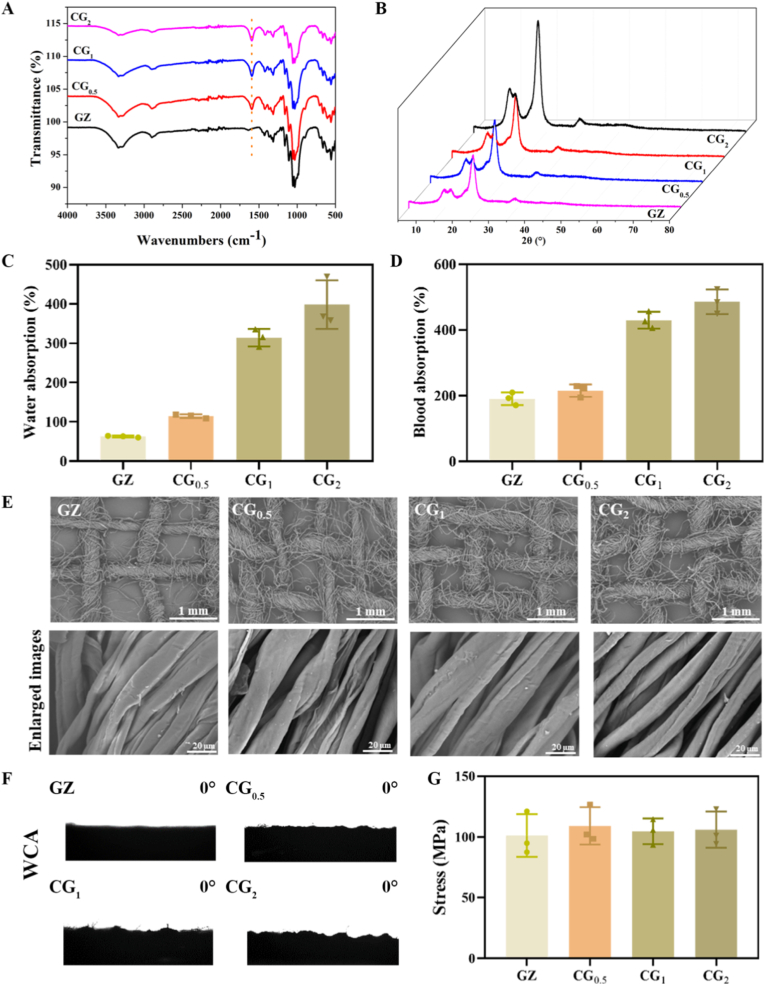
**Characterizations of gauze (GZ) and carboxymethylated gauze (CG).** The FT-IR spectra (A), XRD patterns (B), water absorption rates (C), and blood absorption rates (D) of the GZ and CGs. The SEM images (E) and water contact angles (F) of the GZ and CGs. The rupture stresses of (G) dry GZ and CGs.

To achieve localized release of senolytics at diabetic wound sites, we constructed an asymmetric fabric-based composite platform loaded with ABT-263. As is well known, although the field of hydrogel-based wound dressings has developed rapidly, the commonly used dressings in clinical practice are still CG. Therefore, to prepare asymmetric dressings, GZ was modified into CG, serving as the basis for both hydrogel modification and paraffin hydrophobic modification. As shown in Fig. 4A, through carboxymethylation treatment, GZ was successfully converted into CG. The CGs exhibit a new absorption peak at 1590 cm^−1^, indicating the successful carboxymethylation modification [19]. Meanwhile, the XRD patterns of the CGs show that their crystallinity is lower than that of GZ, which is consistent with our previous research results (Fig. 4B) [20]. The changes in water absorption and blood absorption rates follow a similar trend. As the amount of newly added substances during the carboxymethylation process increases, both the water absorption and blood absorption rates rise (Fig. 4C and D). Additionally, the SEM images shown in Fig. 4F indicate no significant differences between GZ and CGs. However, there are wider gaps between the yarns (Fig. 4E). The water contact angles (WCA) of all CGs and GZ are 0° (Fig. 4F). Fig. 4G presents the rupture stresses of the four kinds of fabrics, which show that there is little difference in the tensile stress of these four materials in the dried state.

**Fig. 5:**
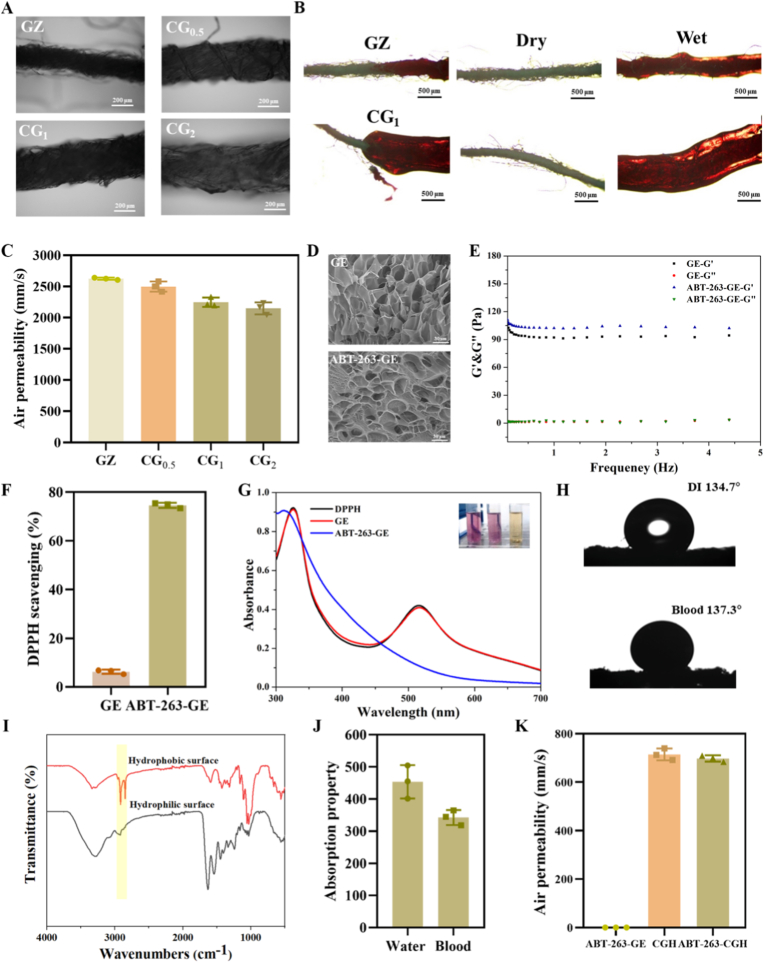
**Characterizations of gelatin hydrogel (GE), ABT-263-loaded hydrogel (ABT-263-GE), ABT-263-GE-loaded GC (CGH), and asymmetric wound dressing (ABT-263-CGH).** (A) OM images of GZ and CGs in the wet state, (B) GZ and CG_1_ after blood treatment, (C) Air permeability of GZ and CGs. (D) SEM images and (E) rheological properties of GE and ABT-263-GE. (F) The DPPH scavenging efficiency of GE and ABT-263-GE. (G) UV–vis spectra of DPPH solutions after reacting with GE and ABT-263-GE. (H) Contact angles of the hydrophobic surface of ABT-263-CGH with water and blood. (I) Infrared spectra of the hydrophilic and hydrophobic surfaces of ABT-263-CGH. (J) Water absorption and blood absorption properties of ABT-263-CGH. (K) Air permeability property of CGH, ABT-263-CGH, and ABT-263-GE.

Fig. 5A shows the images of dry GZ and CG fabrics in their wet state, where the yarns in these fabrics appear similar. However, the wet yarns of CG_2_ show significant expansion, in contrast to GZ and CG_0.5_, which are more similar to each other. Fig. 5B shows blood-treated GZ and CG_1_. In addition to wetting the fibers, blood can also form a blood interface on the surface of CG_1_, which is one of the reasons for the good blood absorption of CG_1_ in the previous figure. In the dry state, GZ, CG_0.5_, and CG_1_ all exhibit excellent gas permeability with little difference between them (Fig. 5C), while CG_2_ shows a slight decrease in gas permeability. Therefore, considering water absorption, blood absorption, permeability, contact angle, and rupture stresses, CG_1_ was selected as the base material for the preparation of asymmetric dressings.

Fig. 5D shows the SEM images of GE and ABT-263-GE hydrogels. From the figure, it can be seen that ABT-263-GE exhibits a porous structure. Fig. 5E shows the rheological properties of GE and ABT-263-GE hydrogels. It can be observed that the hydrogel maintains a relatively stable state with small changes in frequency. The antioxidant activities of GE and ABT-263-GE hydrogels were evaluated using the DPPH scavenging efficiency. As shown in Fig. 5F, the ABT-263-GE hydrogels achieved a DPPH scavenging efficiency of 74.56 %, indicating that the hydrogel has good antioxidant activity. Fig. 5G shows the absorption curve after the reaction between the DPPH solution and the hydrogel. After the addition of ABT-263-GE hydrogel, the original purple color of the DPPH solution almost completely faded. Fig. 5H shows the contact angles of the hydrophobic surface of ABT-263-CGH. The contact angle with water was 134.7°, while with blood, it reached 137.3°. Fig. 5I shows the infrared spectra of the front and back surfaces of ABT-263-CGH. The spectrum indicates the presence of characteristic absorption peaks of paraffin on the hydrophobic surface. Fig. 5J shows the water absorption and blood absorption properties of ABT-263-CGH; the water and blood absorption rates of the composite materials remained above 300 % even after hydrophobic treatment. Fig. 5K shows the permeability characteristics of ABT-263-GE hydrogel, ABT-263-CGH, and pure CGH. It was observed that the air permeability of CGH was comparable to that of the two-faced dressing, while the pure hydrogel exhibited significantly lower air permeability.

Fig. S7A and B show the UV–vis spectrum and calibration curve of ABT-263, and Fig. S7C presents the release curve of ABT-263-GE hydrogel. The release rate of ABT-263 was faster during the first 0–10 h, with the cumulative release rate significantly increasing to 68.94 %, indicating a noticeable drug release behavior in the initial stage. This could be due to the drug diffusing from the surface or easily swollen areas of the hydrogel. After 10 h, the release rate slows down significantly, and from 24 to 48 h, it remains stable, indicating that the drug release from the hydrogel has entered a slow phase, and the system gradually reaches a release equilibrium, demonstrating sustained-release characteristics. The Avrami equation, as a classical kinetic model, is widely used in the study of gas-solid or liquid-solid phase transition mechanisms [21]. Table S3 lists the release kinetics parameters of ABT-263-GE hydrogel calculated based on the Avrami equation. The value of n for ABT-263-GE hydrogel lay between 0 and 0.54, indicating that the release mechanism of ABT-263-GE hydrogel was a composite mechanism of zero-order release and diffusion-limited release.

To evaluate the biosafety of CGH, cytocompatibility assays were performed separately in L929 fibroblasts and HUVECs. For L929 cells, OD values were measured on days 1, 3, 5, and 7 following treatment with CGH extract or culture in complete medium alone. No significant differences in cell viability were observed between the two groups (Fig. S8A). Consistent with these findings, Calcein-AM/PI live/dead staining revealed comparable proportions of viable cells, indicating that CGH extract did not induce cytotoxicity in L929 cells (Fig. S8B). Similarly, in HUVECs, OD measurements showed no significant difference between CGH-treated and control groups throughout the 7-day culture period (Fig. S8C). Live/dead staining further confirmed that the CGH extract had no adverse effect on endothelial cell viability (Fig. S8D). These results collectively demonstrate the good biocompatibility and non-cytotoxic nature of CGH in both fibroblasts and endothelial cells.

### 3.5 ABT-263-CGH effectively eliminates senescent cells in vitro

**Fig. 6:**
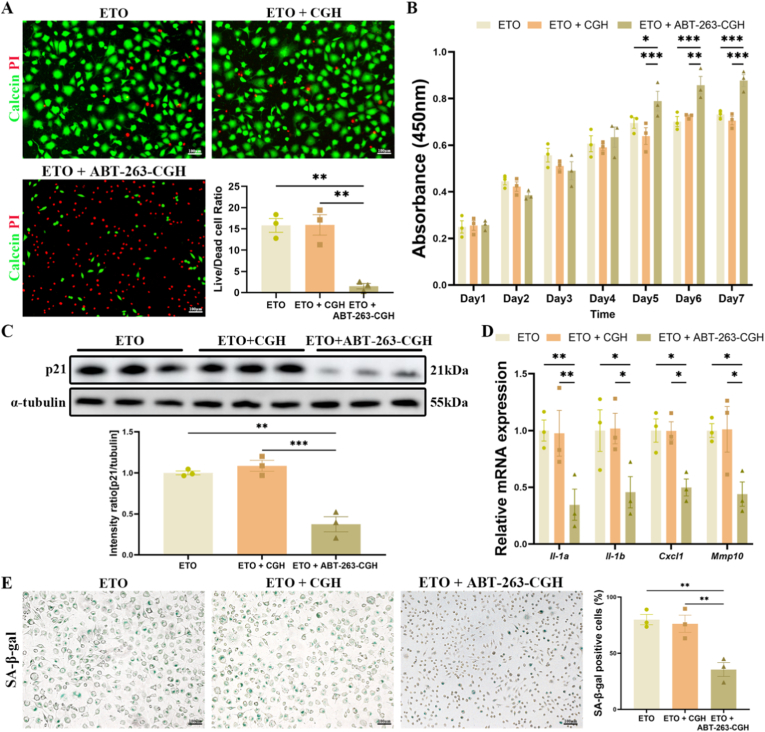
**Evaluation of cell viability and apoptosis following treatment with ABT-263-CGH in senescent L929 cells.** (A) Representative images of Calcein-AM/PI staining used to evaluate cell viability and apoptosis in etoposide (ETO)-induced senescent L929 cells after 48-h treatment with unloaded CGH (n = 3) or ABT-263-CGH (n = 3), compared to untreated controls (ETO, n = 3). Live cells were stained green (Calcein-AM), and dead cells were stained red (Propidium Iodide, PI); the live/dead cell ratio was quantified. (B) Quantitative assessment of cell viability in senescent L929 cells treated with unloaded CGH (n = 3) or ABT-263-CGH (n = 3) for 48 h, compared to untreated controls (ETO, n = 3), measured using the CCK-8 assay over 7 days. (C) Western blot analysis of p21 protein expression in senescent L929 cells after 48-h treatment with unloaded CGH (n = 3) or ABT-263-CGH (n = 3), compared to untreated controls (ETO, n = 3), with densitometric quantification. (D) RT-qPCR analysis of mRNA levels of SASP factors (*Il-1*, *Il-1b*, *Cxcl1*, and *Mmp10*) in senescent L929 cells after treatment (n = 3). (E) Representative images of SA-β-Gal staining in senescent L929 cells treated with unloaded CGH (n = 3) or ABT-263-CGH (n = 3) for 48 h, compared to untreated controls (ETO, n = 3), with quantification of SA-β-Gal-positive cells. Values are expressed as mean ± SEM. Statistical analysis was performed using Student's t-test or one-/two-way ANOVA. ∗P < 0.05, ∗∗P < 0.01, ∗∗∗P < 0.001. (For interpretation of the references to color in this figure legend, the reader is referred to the Web version of this article.)

To evaluate the therapeutic efficacy of ABT-263-CGH, we initially carried out a series of in vitro experiments. Both CGH and ABT-263-CGH extracts were used to culture ETO-induced senescent L929 cells separately, with complete medium serving as the control group. Calcein (live cells, red)/Propidium Iodide (PI, dead cells, green) staining demonstrated that ABT-263-CGH significantly induced apoptosis in SnCs (Fig. 6A). To assess the effect on cell viability, we measured the optical density (OD) at 450 nm daily for seven consecutive days and constructed a cell growth curve. The results revealed that from day 4 onwards, ABT-263-CGH outperformed the control and non-loaded CGH groups, with significant differences becoming apparent starting on day 5 (Fig. 6B). Further investigations showed that ABT-263-CGH effectively cleared SnCs. This was confirmed by a decrease in the relative protein expression levels of p21 (Fig. 6C), a notable decline in the relative mRNA levels of SASP, including *Il-1a*, *Il-1b*, *Cxcl1*, and *Mmp10* (Fig. 6D), and a reduction in the SA-β-gal positive staining rate (Fig. 6E).

To further validate the therapeutic efficacy of ABT-263-CGH, we conducted parallel in vitro experiments using HUVECs. Live/dead cell staining with Calcein (red) and PI (green) indicated substantial induction of apoptosis in senescent HUVECs by ABT-263-CGH (Fig. S9A). The seven-day consecutive cell viability assay revealed a marked growth advantage in the ABT-263-CGH group starting on day 5 (Fig. S9B). Mirroring the observations in L929 cells, ABT-263-CGH significantly reduced the presence of senescent HUVECs, as reflected by downregulated p21 expression at protein levels (Fig. S9C), suppressed expression of the four SASP-related genes (Fig. S9D), and decreased the proportion of SA-β-gal-positive cells (Fig. S9E).

### 3.6 ABT-263-CGH effectively promotes chronic wound healing in diabetic models

To assess the in vivo safety of the CGH dressing, we evaluated liver and kidney function indicators in normal mice with and without a CGH dressing applied to their wounds. Specifically, we measured serum levels of alanine aminotransferase (ALT) and aspartate aminotransferase (AST) as markers of liver function, as well as creatinine (CREA) as a marker of kidney function. All parameters remained within normal ranges (Fig. S10A). Additionally, HE staining of liver and kidney tissues revealed no pathological abnormalities, confirming the dressing's biocompatibility and safety in vivo (Fig. S10B).

**Fig. 7:**
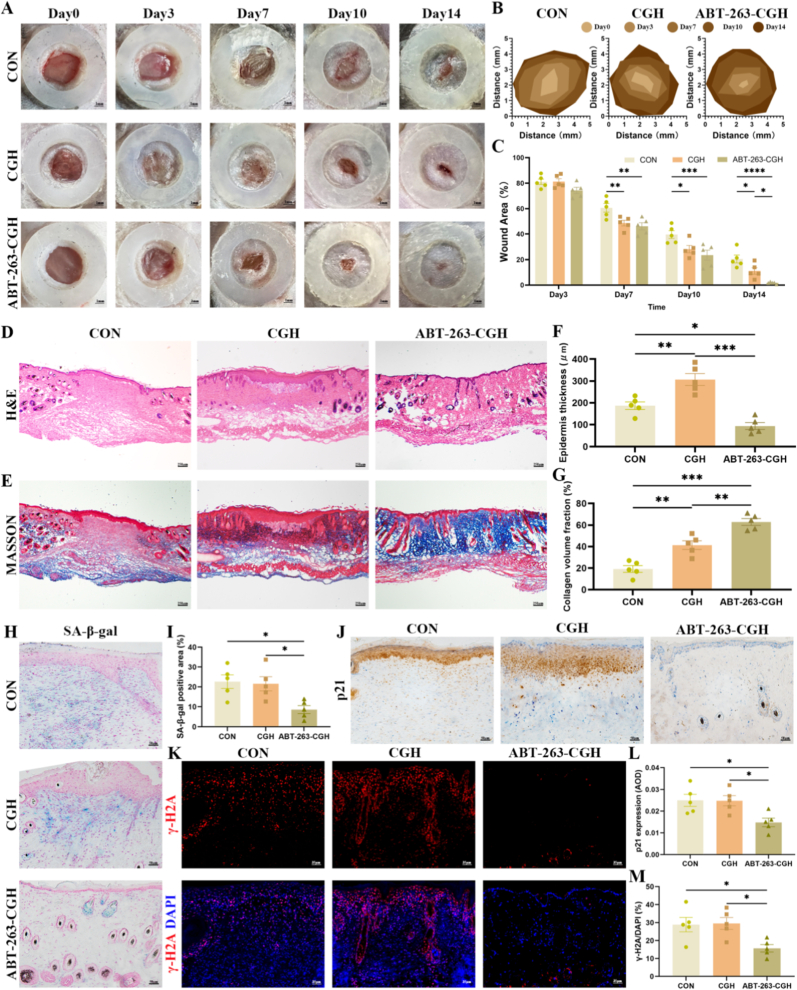
**ABT-263-CGH promotes chronic wound healing in diabetic mice.** (A) Representative wound images on days 0, 3, 7, 10, and 14 post-injury from diabetic mice receiving no dressing (CON, n = 5), unloaded CGH (n = 5), or ABT-263-CGH (n = 5). (B–C) Wound healing progression and wound area quantification over 14 days in the three treatment groups. (D–E) Representative H&E and Masson's trichrome staining images of wound tissues collected on day 14 from diabetic mice receiving no dressing (CON, n = 5), unloaded CGH (n = 5), or ABT-263-CGH (n = 5). (F–G) Quantification of epidermal thickness and collagen area based on histological analyses from diabetic mice receiving no dressing (CON, n = 5), unloaded CGH (n = 5), or ABT-263-CGH (n = 5). (H–I) Representative SA-β-Gal staining images of wound sections and quantification of SA-β-Gal-positive cells from diabetic mice receiving no dressing (CON, n = 5), unloaded CGH (n = 5), or ABT-263-CGH (n = 5). (J, L) Representative immunohistochemical staining images and quantification (AOD method) of p21 expression in wound tissues from diabetic mice receiving no dressing (CON, n = 5), unloaded CGH (n = 5), or ABT-263-CGH (n = 5). (K, M) Representative immunofluorescence staining images of γ-H2AX and quantification of γ-H2AX-positive nuclei (γ-H2AX/DAPI) from diabetic mice receiving no dressing (CON, n = 5), unloaded CGH (n = 5), or ABT-263-CGH (n = 5). Values are presented as mean ± SEM. Statistical analysis was performed using one- or two-way ANOVA. ∗P < 0.05, ∗∗P < 0.01, ∗∗∗P < 0.001.

To further investigate the impact of this material on chronic wound healing in diabetic conditions, we conducted a series of in vivo experiments. We created a full-thickness 5 mm punch wound on STZ-induced diabetic mice, which were randomly divided into three groups: a local application of CGH, ABT-263-CGH, and a control group with no dressing. To prevent skin contraction, we secured the wound with adhesive glue and a pad ring. Imaging was performed on days 0, 3, 7, 10, and 14. The results revealed that by day 14, ABT-263-CGH had almost completely healed the wound, while the control and CGH groups showed significant non-healed areas (Fig. 7A and B). Analysis of the wound area demonstrated that from day 7 onwards, both the CGH and ABT-263-CGH groups significantly promoted chronic wound healing. However, ABT-263-CGH showed a faster rate of wound closure, with its healing effect on day 14 significantly superior to that of CGH alone (Fig. 7C). On day 14, histological analysis through H&E and Masson trichrome staining revealed that in the control group, the wound had not yet formed significant granulation tissue, with minimal collagen deposition and the initiation of epithelial regeneration, indicating a transition from the inflammatory phase to the proliferative phase. In the CGH group, notable epithelialization and granulation tissue formation were observed, with collagen synthesis beginning and inflammation gradually subsiding, characteristic of the proliferative phase. In the ABT-263-CGH group, there was extensive collagen deposition with increasing organization, sparse inflammatory cell infiltration, and epithelial thinning with nearly complete healing, indicating progression into the collagen remodeling and tissue maturation phase (Fig. 7D–G). These findings suggest that this drug-loaded material significantly enhances chronic wound healing. Additionally, SnCs were effectively cleared in the ABT-263-CGH group, as demonstrated by SA-β-gal staining (Fig. 7H and I), p21 immunohistochemistry (Fig. 7J and L), and γ-H2AX immunofluorescence staining (Fig. 7K and M), indicating that the material promotes wound healing by targeting and eliminating SnCs.

To further evaluate the durability of wound healing, we performed a long-term follow-up until day 42 post-injury. In the ABT-263-CGH group, all wounds remained completely closed without any evidence of re-ulceration, demonstrating stable and sustained repair beyond the early healing phase (Fig. S11A). Moreover, functional assessments at day 42 showed significantly improved skin hydration and elasticity compared with controls, indicating effective restoration of barrier integrity and mechanical resilience (Fig. S11B and C). Histological analyses (H&E and Masson) further confirmed full re-epithelialization, well-organized collagen deposition, and partial regeneration of hair follicles in the ABT-263-CGH group (Fig. S11D–G). Collectively, these results highlight that ABT-263-CGH not only accelerates structural repair but also promotes functional recovery of regenerated skin, supporting its long-term therapeutic efficacy in diabetic wound healing.

## 4 Discussion

Cellular senescence plays a critical role in the chronicity of diabetic wounds [22]. In this study, we employed multiple senescence markers in both human and murine tissues, together with single-cell transcriptomic analysis of DFU patient samples, to delineate senescence expression profiles across diverse cell types, including fibroblasts and endothelial cells. Moreover, we developed an asymmetric fabric-based hydrogel platform as a localized senolytic delivery system to promote wound healing in diabetic chronic wounds, providing a novel and clinically promising therapeutic strategy for senescence-targeted treatment of chronic diabetic wounds.

Previous studies have demonstrated the presence of senescent cells in DFUs [23]. However, given the heterogeneity of senescent cells and the specificity of senescence markers [24], this study adhered to recently published guidelines for detecting senescent cells by employing at least three markers (p21/p16, γ-H2AX, and SA-β-Gal) for staining and quantitative analysis across multiple independent samples [25,26], thereby comprehensively characterizing senescent cell enrichment in chronic diabetic wounds. Notably, we integrated the SenMayo gene set with key senescence markers (p21) and apoptosis-related genes (Bcl-2 family members) for single-cell transcriptomic analysis of human DFU samples, which revealed pronounced senescence in fibroblasts accompanied by elevated anti-apoptotic gene expression. This analysis revealed pronounced senescence in fibroblasts, accompanied by elevated expression of anti-apoptotic genes. Similarly, Wei et al. reported that resistance to ferroptosis, another form of programmed cell death, may contribute to the high burden of senescent fibroblasts in diabetic wounds [27]. Our fibroblast subcluster analysis further identified senescent fibroblasts predominantly within pro-inflammatory subsets, providing a theoretical basis for targeted interventions aimed at senescence in chronic wounds.

Histological staining further revealed positive expression of p21 and p16 in the papillary and reticular dermis, with stronger and more localized signals in the epidermal–dermal junction (EDJ) area. We hypothesize that this spatial distribution reflects increased sensitivity of cells in the EDJ region (such as superficial dermal fibroblasts) to chronic stressors such as hyperglycemia, oxidative stress, and inflammation, leading to activation of p21-and p16-mediated cell cycle arrest and senescence pathways [28]. This observation is consistent with previous reports noting enrichment of p16-positive cells at the EDJ in diabetic chronic wounds, potentially due to the susceptibility of these cells to damage and impaired clearance of senescent cells in this area [29]. Although staining of SA-β-Gal and γ-H2AX was more evenly distributed, this likely reflects differences in the temporal dynamics and functional roles of these markers, with p21 and p16 marking cell cycle regulation and stress response initiation, while SA-β-Gal and γ-H2AX indicate senescence maintenance and DNA damage, respectively, leading to distinct spatial expression patterns [30].

The induction of cellular senescence in DFUs is thought to involve hyperglycemia-induced oxidative stress, chronic inflammation, accumulation of AGEs, and local ischemia [5]. These factors contribute to DNA damage and metabolic dysregulation, inducing senescence in multiple cell types, including fibroblasts, endothelial cells, and macrophages [31]. Importantly, senescent cells in diabetic wounds exhibit enhanced resistance to apoptosis and sustained secretion of pro-inflammatory factors (SASP), forming a detrimental feedback loop that promotes their persistence, exacerbates chronic inflammation, and impedes wound repair [32]. In this study, we employed ETO-induced persistent DNA damage to model stress-induced senescence and established an AGEs-induced fibroblast senescence model to better mimic the chronic hyperglycemic environment of diabetic tissues. AGEs accumulate extensively in diabetic tissues and activate oxidative stress and inflammation through RAGE signaling, representing a key mechanism underlying diabetes-associated tissue aging [33]. However, it is acknowledged that in vitro senescence models differ from in vivo senescent cells in DFU tissues, and other induction methods such as γ-irradiation, which causes acute DNA damage, may not fully recapitulate the chronic metabolic stress-driven senescence observed in diabetic wounds [34]. Additionally, γ-irradiation requires specialized equipment, precise dose control, and may induce extensive cell death [35].

Given that the accumulation of senescent cells is a key contributor to chronic wound development and persistence, targeting cellular senescence has emerged as a promising therapeutic approach [36]. Senolytics, initially developed for aging-related diseases, selectively eliminate apoptosis-resistant senescent cells and are increasingly being investigated in wound healing contexts [37]. Samarawickrama et al. utilized the senescence-detecting probe XZ1208 to demonstrate that delayed wound healing in diabetic mice correlates with senescent cell accumulation at wound sites, and showed that repeated intraperitoneal injections of ABT-263 effectively clear senescent cells and accelerate wound closure [38]. Shvedova et al. further confirmed that topical pre-application of ABT-263 reduces senescence marker expression and promotes wound healing, although it transiently induces inflammation and macrophage infiltration in aged mouse skin [39]. In our study, the ABT-263-loaded fabric hydrogel achieved localized drug enrichment and sustained release, enhancing therapeutic efficacy while significantly reducing systemic toxicity. ABT-263, a well-studied Bcl-2/Bcl-xL inhibitor, exerts senolytic effects largely by exploiting senescent cells' dependence on anti-apoptotic signaling, showing selective cytotoxicity toward senescent cells within an appropriate dose range [40]. Our in vitro drug screening confirmed lower toxicity of ABT-263 toward normal fibroblasts compared to senescent fibroblasts at the applied doses. The controlled release properties and mild environment (neutral pH, gradual release) of the CGH platform reduce local peak drug concentrations, further improving senescent cell targeting and minimizing off-target effects on healthy tissues. In vivo, wounds treated with ABT-263-CGH healed significantly faster without histological abnormalities, excessive inflammation, or notable toxicity, indirectly suggesting minimal adverse effects on normal cells. Collectively, these results indicate that the current formulation and dosing confer favorable selectivity and biocompatibility, efficiently clearing senescent cells while sparing non-senescent cells.

Hydrogels have gained popularity as wound dressings due to their excellent biocompatibility, mechanical properties, biodegradability, and moisture retention [41,42]. Consistent with these reports, CGH alone also promoted wound healing in our study. Owing to their biocompatibility and sustained-release capabilities, hydrogels are ideal for drug delivery, enabling controlled release, reduced dosing frequency, and improved patient compliance [43,44]. Liu et al. developed a ROS/glucose-responsive quercetin-loaded hydrogel with antibacterial, antioxidant, anti-inflammatory, and pro-angiogenic properties that accelerated diabetic wound healing [45]. Similarly, Zhang et al. created a quercetin-loaded keratin/carboxymethyl β-cyclodextrin/polyurethane hydrogel that enhanced quercetin's water solubility, bioavailability, and sustained release, exhibiting strong antioxidant and antibacterial effects and promoting cell proliferation, migration, angiogenesis, and wound remodeling [46]. By combining functional hydrogels with cotton gauze, we developed an asymmetric composite dressing with improved water absorption, humidity control, and air permeability. The asymmetric design enables the hydrophilic side of the dressing to effectively load and sustain the release of ABT-263, achieving localized drug enrichment and controlled release at the wound site, thereby significantly improving local bioavailability while reducing systemic toxicity risk. The hydrophobic side functions as a barrier against external contamination, ensuring a stable wound environment. The porous structure of the cotton gauze significantly enhances the breathability of the dressing, facilitating the maintenance of a moist yet non-occlusive healing microenvironment, while the hydrogel component provides structural support for cell adhesion and proliferation, thereby promoting tissue repair. Using a diabetic chronic wound model, we demonstrated accelerated re-epithelialization, increased collagen deposition, and reduced senescent cell accumulation. The design and application of this asymmetric composite dressing not only optimized the therapeutic effects of ABT-263 but also provided new theoretical insights and translational potential for the combined clearance of senescent cells and tissue repair in diabetic wounds such as DFUs. Furthermore, the ABT-263-CGH possesses excellent versatility and potential for broader applications. Its favorable flexibility, biocompatibility, and controlled release properties suggest applicability beyond diabetic chronic wounds to other refractory chronic wounds such as burns, pressure ulcers, and vascular ulcers [47]. Therefore, this platform holds promise as a universal localized senolytic therapy with potential in broader dermatological regenerative medicine.

One limitation of this study is its primary focus on the senescence characteristics of fibroblasts within the wound microenvironment and the effects of ABT-263-CGH on targeted clearance of senescent cells and tissue repair. Therefore, a systematic analysis of immune cell populations such as macrophages and T cells was not performed. However, H&E staining showed reduced inflammation and better tissue integrity in treated wounds, suggesting possible immunomodulatory effects. Considering that senescent cells secrete numerous chemokines and inflammatory mediators via SASP, driving aberrant immune cell infiltration, clearance of senescent cells may partially improve the local immune milieu in wounds [48]. Future studies combining immunohistochemistry or flow cytometry will systematically assess the impact of ABT-263-CGH on immune cell composition and function during wound healing to further elucidate its immunoregulatory potential. Another limitation is that single-cell sequencing samples and dissociation methods were derived from whole wound tissues, lacking fine spatial resolution to reconstruct EDJ structures. Future integration with spatial transcriptomics is needed to clarify spatial-transcriptional characteristics of senescent cells in distinct regions. Lastly, this study was primarily conducted at the cellular and animal levels, with no human trials performed. Clinical translation remains challenging, underscoring the urgent need for further preclinical and clinical studies to rigorously validate both efficacy and safety.

## 5 Conclusion

In conclusion, our study explores the potential of fabric-based asymmetric wound dressing loaded with senolytic agents, specifically ABT-263, to enhance chronic wound healing. By integrating materials science, cell biology, and animal science, we evaluated the efficacy and safety of this novel therapeutic approach in both in vitro and in vivo models. Our findings demonstrate the feasibility of this strategy in promoting wound healing by targeting the burden of SnCs in chronic diabetic wounds. By targeting cellular senescence, this dressing can enhance tissue regeneration and promote faster, more efficient wound closure. However, successful clinical translation will require further preclinical and clinical studies to determine optimal dosage, safety profiles, and long-term efficacy. Future research should also focus on optimizing the hydrogel formulation, characterizing the release kinetics of senolytic agents, and validating their effectiveness in various settings, such as by enhancing responsiveness to environmental stimuli like pH, light, and heat through the combination with other biomolecules or upgrading to a smart gel system. This innovative approach has the potential to revolutionize chronic wound management by addressing the cellular mechanisms underlying impaired healing, ultimately improving patient outcomes and reducing the healthcare burden associated with chronic wound care.

## CRediT authorship contribution statement

**Ming Zhang:** Writing – original draft, Visualization, Methodology, Investigation, Formal analysis. **Yamei Wang:** Writing – original draft, Visualization, Validation, Methodology, Investigation, Formal analysis. **Yao Dai:** Software, Formal analysis, Data curation. **Yan Hu:** Software, Formal analysis, Data curation. **Wanting Fu:** Software. **Yuhao Zhao:** Software. **Hanyu Ma:** Software. **Di Zhang:** Software. **Ying Chen:** Software. **Yixuan Zhou:** Software. **Lei Du:** Funding acquisition. **Jing Chang:** Funding acquisition. **Fang Liu:** Funding acquisition. **Shuyan Chen:** Supervision, Resources, Project administration. **Fei Wang:** Supervision, Resources, Project administration. **Dongdong Xiao:** Methodology, Investigation. **Zhen Li:** Writing – review & editing, Visualization, Funding acquisition, Conceptualization.

## Declaration of competing interest

The authors declare that they have no known competing financial interests or personal relationships that could have appeared to influence the work reported in this paper.

## Data Availability

Data will be made available on request.
