# Supplementary material for: Senolytic-loaded asymmetric wound dressing for targeted senescent cell clearance in diabetic wound healing
Source: Mater Today Bio. 2025 Dec 24;36:102741. doi: 10.1016/j.mtbio.2025.102741 (PMC12813340; doi:10.1016/j.mtbio.2025.102741)
Supplement: Multimedia component 1 [file mmc1.pdf]

Supplementary Materials

Senolytic-Loaded Asymmetric Wound Dressing for Targeted Senescent Cell Clearance in Diabetic Wound Healing

Ming Zhang [a,1], Yamei Wang [b,1], Yao Dai [a,1], Yan Hu [a,1], Wanting Fu [c,1], Yuhao Zhao [a], Hanyu Ma [a], Di Zhang [a], Ying Chen [a], Yixuan Zhou [d], Lei Du [a], Jing Chang [a], Fang Liu [a], Shuyan Chen [a,*], Fei Wang [a,*], Dongdong Xiao [e,*], Zhen Li [a,*]

[a] Department of Geriatrics, Xinhua Hospital, Shanghai Jiao Tong University School of Medicine, Shanghai, China.

[b] School of Perfume and Aroma Technology, Shanghai Institute of Technology, Shanghai, China.

[c] Department of Pharmacy, Xinhua Hospital, Shanghai Jiao Tong University School of Medicine, Shanghai, China.

[d] Department of Cardiovascular Surgery, Shanghai General Hospital, Shanghai, China.

[e] Department of Urology, Ren Ji Hospital, Shanghai Jiao Tong University School of Medicine, Shanghai, China.

[1] These authors contributed equally to this work.

* Corresponding authors.

E-mail addresses: chenshuyan@xinhuamed.com.cn (S. Chen), wangfei01@xinhuamed.com.cn (F. Wang), xiaodd888878@163.com (D. Xiao), lizhen8367@xinhuamed.com.cn (Z. Li).

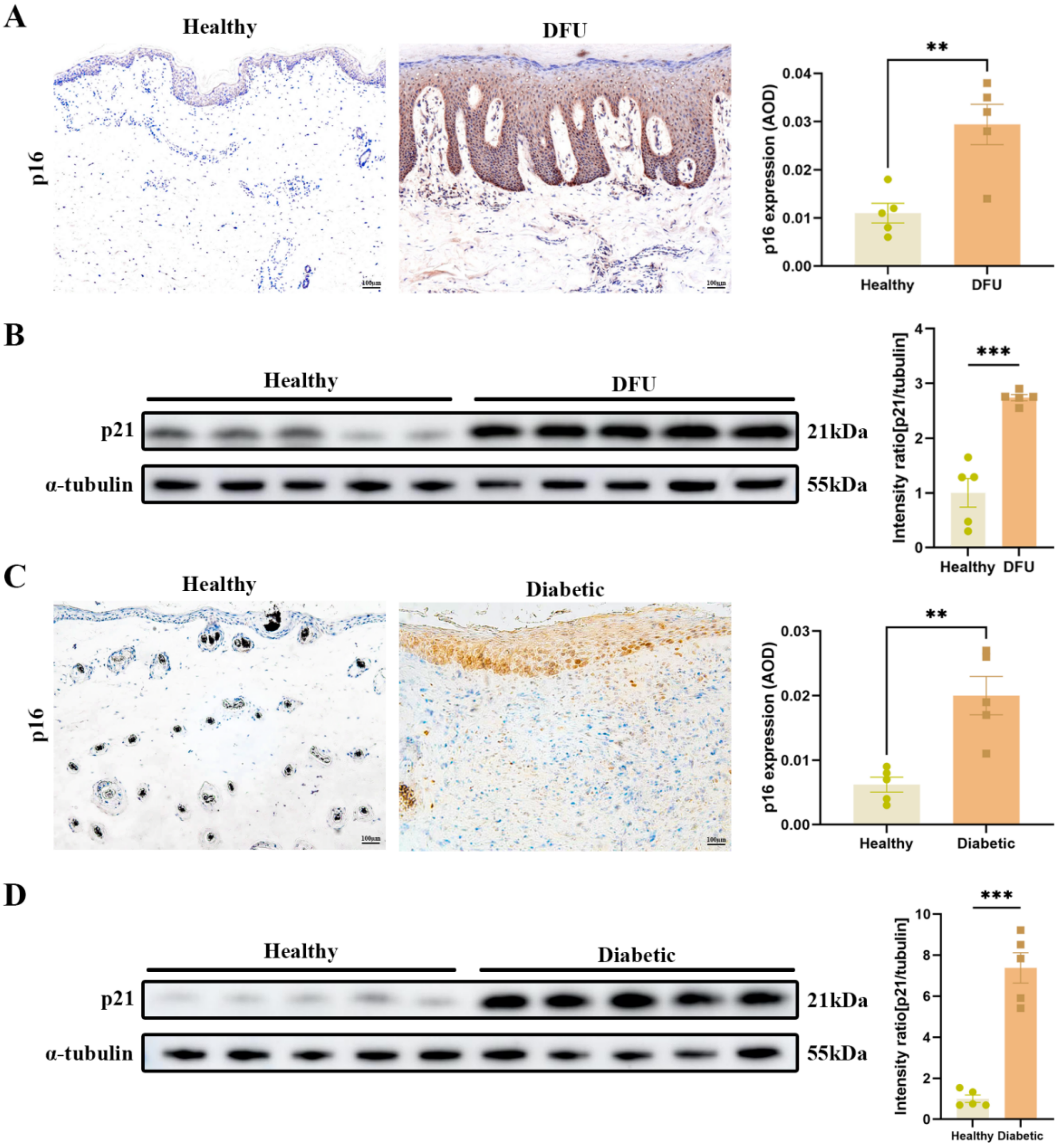

Figure S1. Cellular senescence markers are increased in chronic diabetic wounds. (A) Representative images of immunohistochemical staining of p16 from healthy individuals (n = 5) and DFU patients (n = 5) and quantification (AOD method). (B) Western blot analysis of p21 protein expression in healthy individuals (n = 5) and DFU patients (n = 5). (C) Representative images of immunohistochemical staining of p16 from healthy controls (n = 5) and diabetic wound model mice (n = 5) and quantification (AOD method). (D) Western blot analysis of p21 protein expression in healthy controls (n = 5) and diabetic wound model mice (n = 5). Values are presented as mean ± SEM. Statistical analysis was performed using Student's t-test ANOVA. P < 0.05, P < 0.01, *P < 0.001

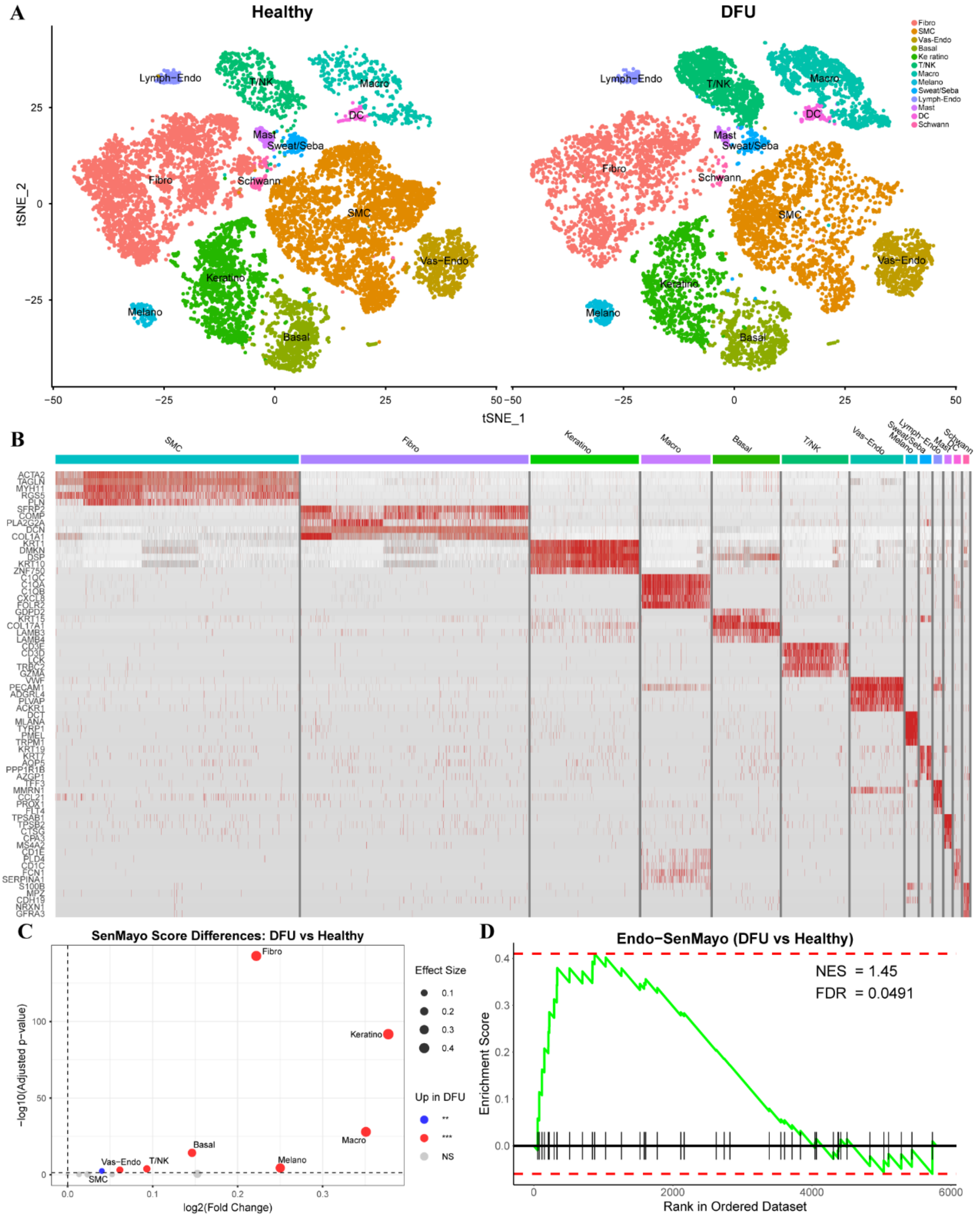

Figure S2. Characterization of cell types and senescence signatures in DFU skin by single-cell RNA sequencing. (A) t-SNE plots of identified cell types in healthy and DFU samples. (B) Violin plots confirming cell-type identities with marker gene expression. (C) Boxplots comparing SenMayo scores in each cell type between groups; eight types show significant increases in DFU ($p < 0.01$). (D) GSEA plot showing significant enrichment of SenMayo genes in DFU-derived endothelial cells (NES = 1.45, FDR < 0.05).

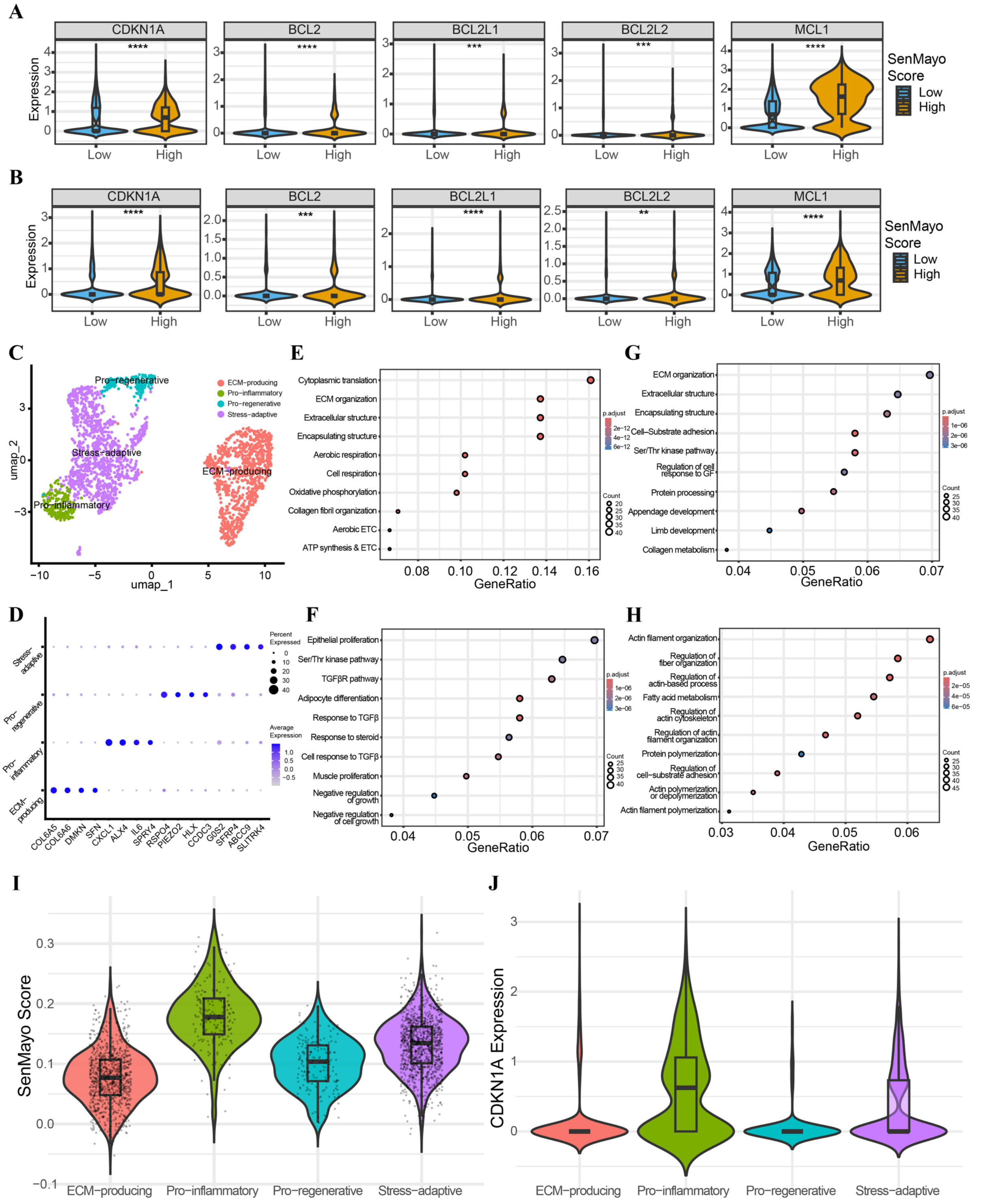

Figure S3. Fibroblast subtypes and senescence-associated gene expression in chronic wounds. (A) Expression of senescence- and anti-apoptosis-related genes in cells with high versus low SenMayo scores. (B) Expression of senescence- and anti-apoptosis-related genes in fibroblasts with high versus low SenMayo scores. (C) UMAP visualization of identified fibroblast subtypes. (D) Dot plot showing representative marker genes for each fibroblast subtype. (E–H) Gene Ontology (GO) Biological Process enrichment for individual

fibroblast subtypes: (E) ECM-producing, (F) pro-inflammatory, (G) pro-regenerative, and (H) stress-adaptive. (I) Distribution of SenMayo scores across fibroblast subtypes. (J) CDKN1A (p21) expression across fibroblast subtypes.

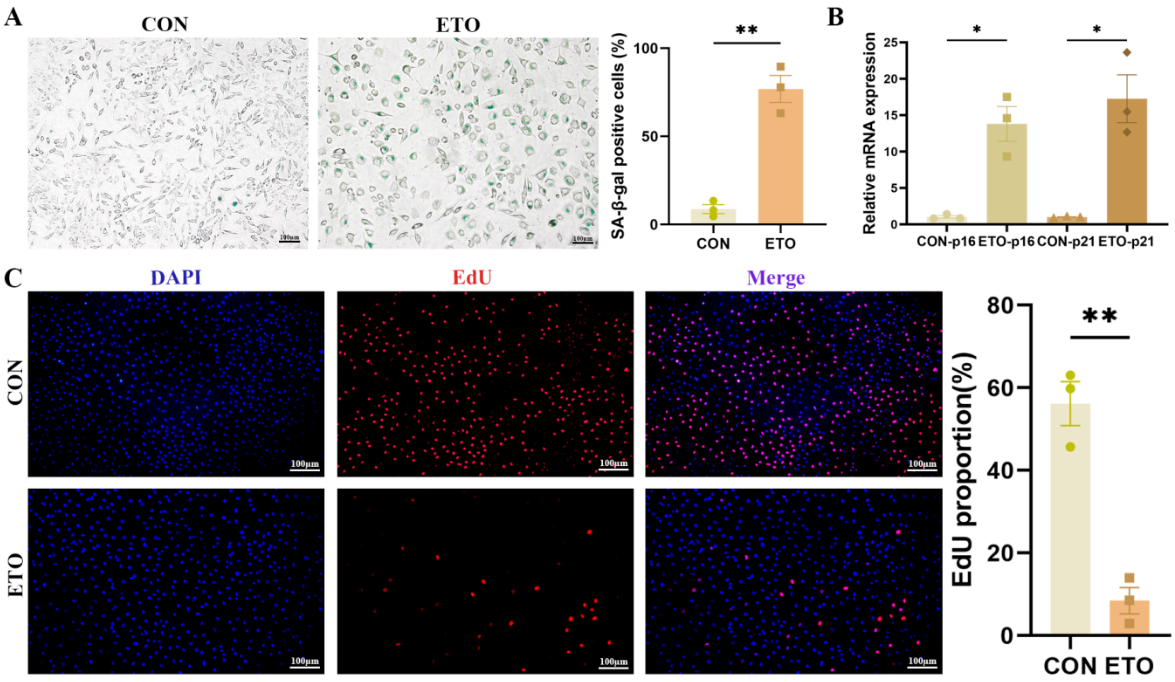

Figure S4. Etoposide (ETO) induces senescence in L929 cells. (A) Representative images of SA-β-gal staining in vehicle-treated (n = 3) and ETO-treated cells (n = 3). (B) qPCR analysis of p16 and p21 expression in vehicle-treated (n = 3) and ETO-treated cells (n = 3). (C) Representative images of EdU incorporation assay in vehicle-treated (n = 3) and ETO-treated cells (n = 3), with quantification of proliferation rates. Values represent mean ± SEM. Statistical analysis was performed using Student's t-test or two-way ANOVA. P < 0.05, P < 0.01, *P < 0.001.

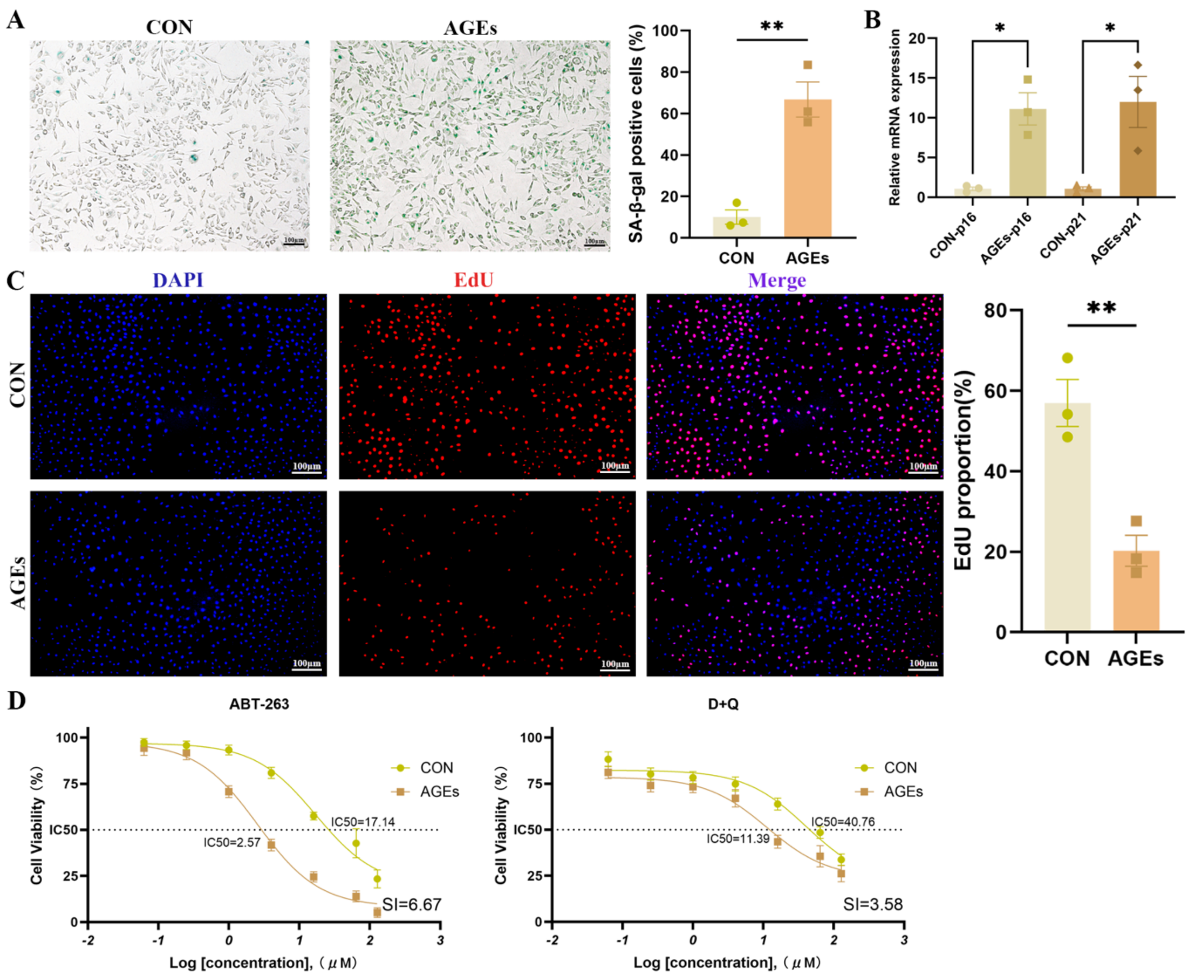

Figure S5. Advanced glycation end products (AGEs) induces senescence in L929 cells. (A) Representative images of SA-β-gal staining in vehicle-treated (n = 3) and AGEs-treated cells (n = 3). (B) qPCR analysis of p16 and p21 expression in vehicle-treated (n = 3) and AGEs-treated cells (n = 3). (C) Representative images of EdU incorporation assay in vehicle-treated (n = 3) and AGEs-treated cells (n = 3), with quantification of proliferation rates. Values represent mean ± SEM. (D) Cell viability assays were performed on vehicle-treated (CON, n = 3) and AGEs-treated (n = 3) L929 cells exposed to varying concentrations of ABT-263 and D+Q for 48 hours. Cell viability was assessed using CCK-8 and expressed as a percentage relative to untreated controls. Dose-response curves and IC_{50} values were calculated for each compound. The selectivity index (SI) was determined by dividing the IC_{50} in proliferating cells by that in senescent cells. Statistical analysis was performed using Student's t-test or two-way ANOVA. P < 0.05, P < 0.01, *P < 0.001.

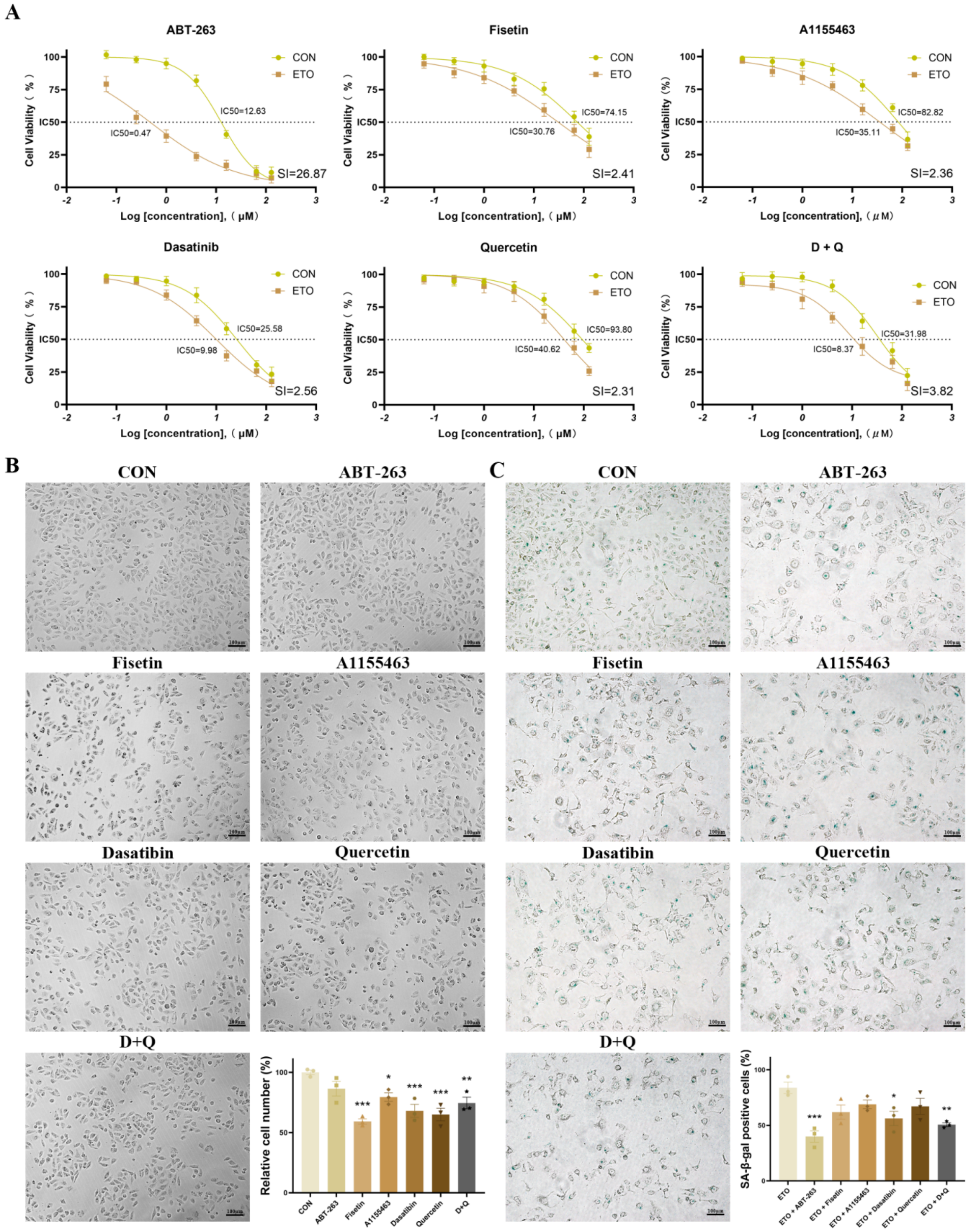

Figure S6. Screening and evaluation of senolytic agents in vehicle-treated and etoposide (ETO)-treated human umbilical vein endothelial cells (HUVECs). (A) Cell viability assay of vehicle-treated (CON, n = 3) and ETO-treated (n = 3) HUVECs exposed to varying concentrations of ABT-263, Fisetin, A1155463, Dasatinib, Quercetin, and D+Q for 48 hours. Cell viability was assessed using CCK-8 and expressed as a percentage relative to untreated controls. Dose-response curves and IC_{50} values were calculated for each

compound. The selectivity index (SI) was determined by dividing the IC_{50} in proliferating cells by that in senescent cells. (B) Cytotoxicity of each senolytic agent (n = 3 for each agent), shown with representative images, at its optimal concentration after 48-hour treatment of vehicle-treated HUVECs, assessed via cell count quantification. (C) Representative images of SA-β-Gal staining of ETO-treated senescent HUVECs treated with each senolytic agent (n = 3 for each agent) at its optimal concentration for 48 hours, with quantification of SA-β-Gal-positive cell percentages. Values represent mean ± SEM. Statistical analysis was performed using Student's t-test or one-/two-way ANOVA. P < 0.05, P < 0.01, *P < 0.001.

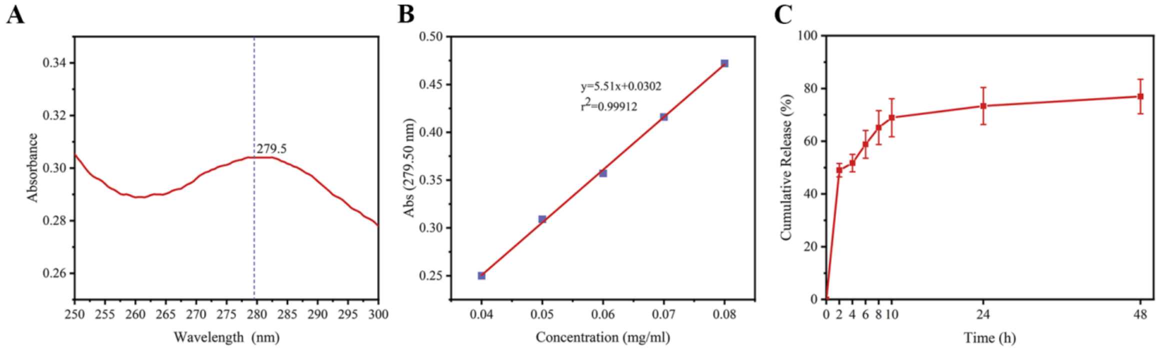

Figure S7. UV–vis characterization and release profile of ABT-263 from ABT-263-GE hydrogel. (A) The UV-vis spectrum and (B) calibration curve of ABT-263, and (C) the release curve of ABT-263-GE hydrogel.

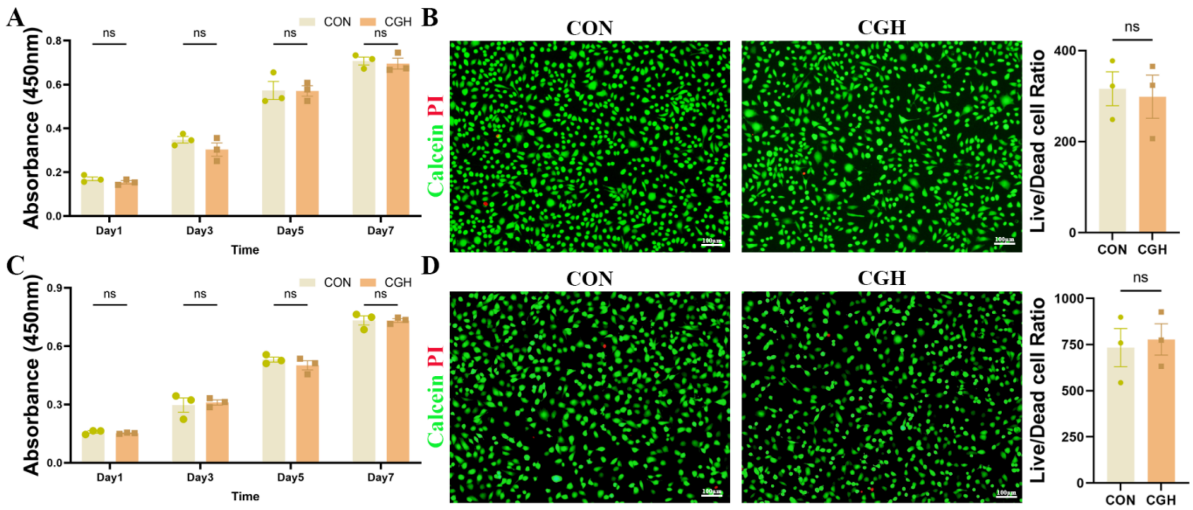

Figure S8. In vitro safety evaluation of CGH in L929 and HUVECs. (A) CCK-8 assay to assess viability of L929 cells treated with CGH extract (n = 3) versus vehicle (CON, n = 3) on days 1, 3, 5, and 7. (B) Calcein-AM/PI staining of L929 cells after 48-hour treatment with CGH extract (n = 3) or vehicle (CON, n = 3), with quantification of the live/dead ratio. (C) CCK-8 assay to assess viability of HUVECs treated with CGH extract (n = 3) versus vehicle (CON, n = 3) on days 1, 3, 5, and 7. (D) Calcein-AM/PI staining of HUVECs after 48-hour treatment with CGH extract (n = 3) or vehicle (CON, n = 3), with quantification of the live/dead ratio. Values are expressed as mean ± SEM. Statistical analysis was performed using Student's t-test or one-/two-way ANOVA. *P < 0.05, **P < 0.01, ***P < 0.001.

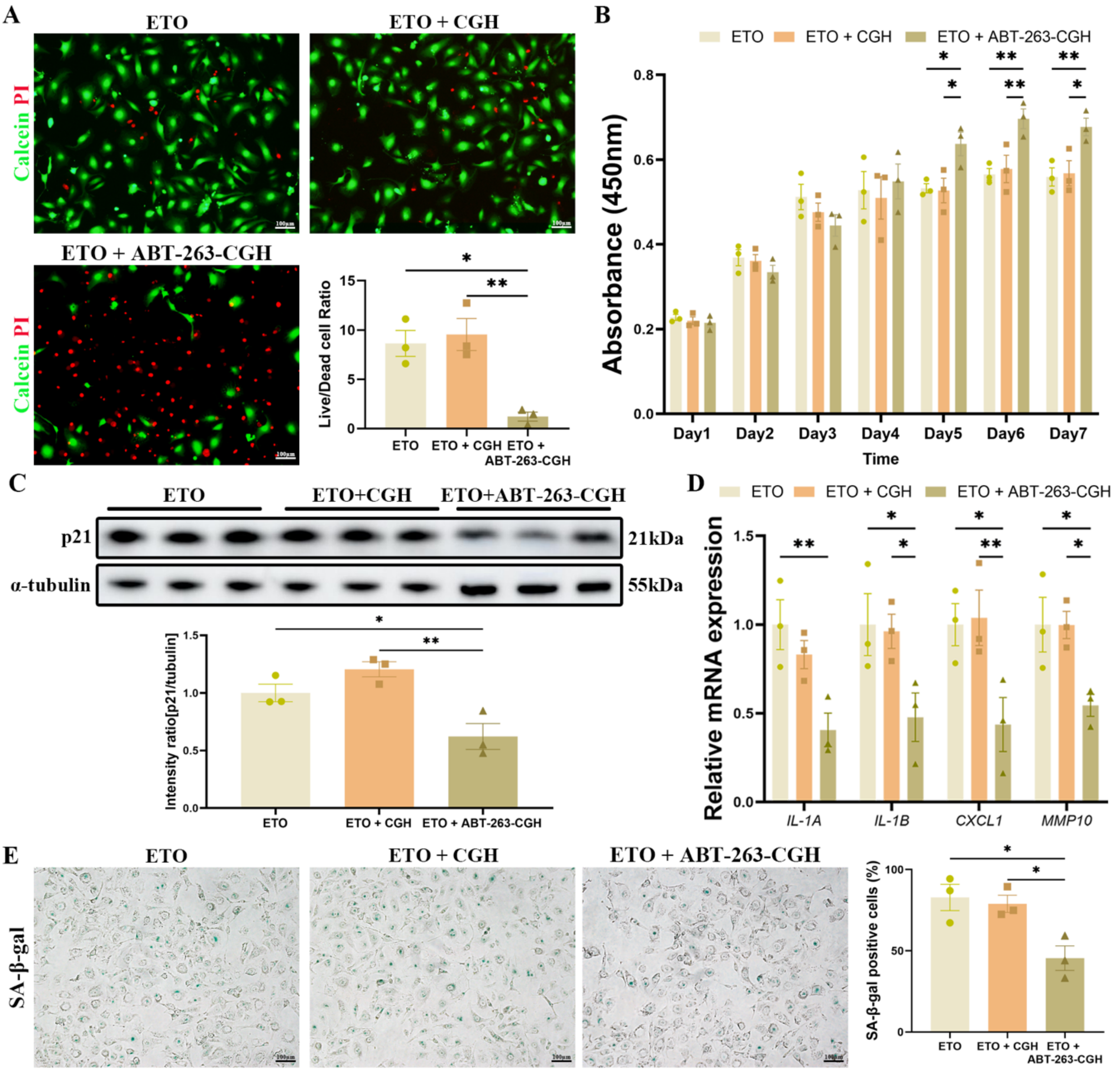

Figure S9. Evaluation of cell viability and apoptosis following treatment with ABT-263-CGH in senescent human umbilical vein endothelial cells (HUVECs). (A) Calcein-AM/PI staining to evaluate cell viability and apoptosis in etoposide (ETO)-induced senescent HUVECs after 48-hour treatment with unloaded CGH (n = 3) or ABT-263-CGH (n = 3), compared to untreated controls (ETO, n = 3). Live cells were stained red (Calcein-AM), and dead cells were stained green (Propidium Iodide, PI); the live/dead cell ratio was quantified. (B) Quantitative assessment of cell viability in senescent HUVECs treated with unloaded (n = 3) or ABT-263-CGH (n = 3) for 48 hours, measured via CCK-8 assay over 7 days. (C) Western blot analysis of p21 protein expression in senescent HUVECs following 48-hour treatment with unloaded (n = 3) or ABT-263-CGH (n = 3), with densitometric quantification. (D) RT-qPCR analysis of mRNA levels of senescence-associated secretory phenotype (SASP) factors (*IL-1A, IL-1B, CXCL1, MMP10*) following treatment (n = 3 for each group). (E) SA-β-Gal staining of senescent HUVECs after treatment with unloaded

(n = 3) or ABT-263-CGH (n = 3) for 48 hours, with quantification of SA-β-Gal-positive cells. Values are expressed as mean ± SEM. Statistical analysis was performed using Student's t-test or one-/two-way ANOVA. P < 0.05, P < 0.01, *P < 0.001.

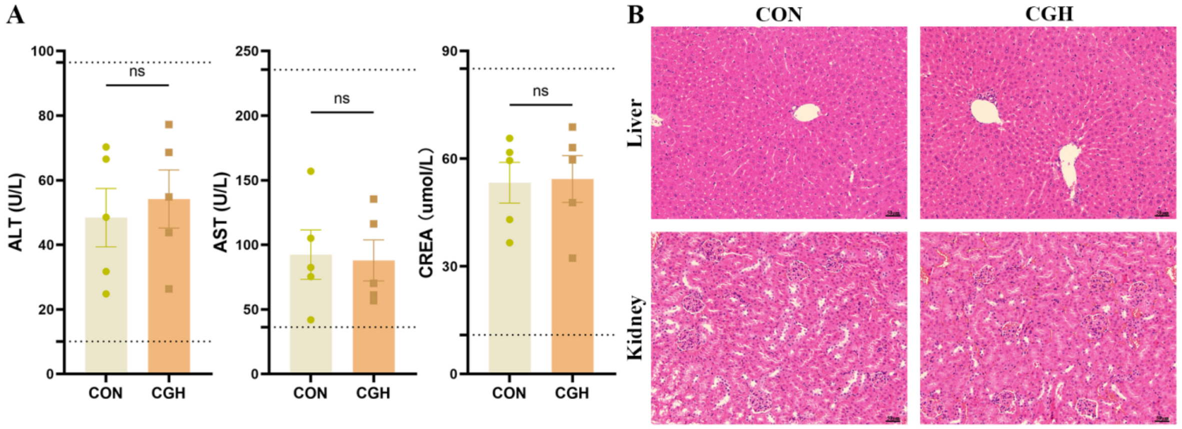

Figure S10. Biosafety evaluation of the CGH dressing in vivo. (A) Serum levels of alanine aminotransferase (ALT), aspartate aminotransferase (AST), and creatinine (CREA) in normal mice without dressing (control, n = 5) and in mice treated with CGH dressing (n = 5) applied to full-thickness skin wounds. Dashed lines indicate the upper and lower limits of normal reference ranges for each biomarker. (B) Representative H&E staining of liver and kidney tissues from control (n = 5) and CGH-treated mice (n = 5). Values are presented as mean ± SEM. Statistical analysis was performed using Student's t-test. ns, not significant.

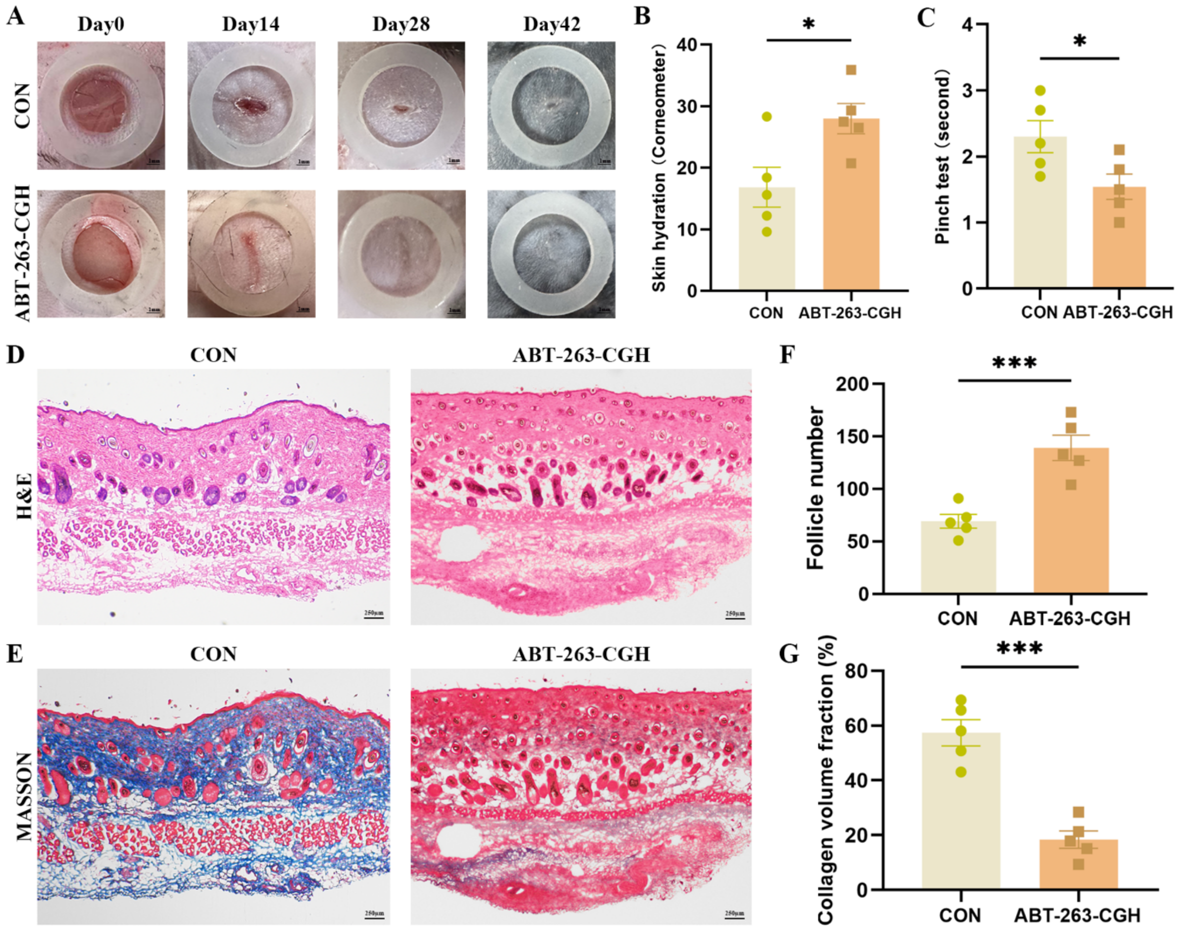

Figure S11. Functional recovery and long-term stability of wound healing in ABT-263-CGH-treated skin. (A) Representative wound images on days 0, 14, 28, and 42 post-injury from diabetic mice receiving no dressing (CON, n = 5) or ABT-263-CGH (n = 5). (B) Skin hydration levels of CON (n = 5) and ABT-263-CGH (n = 5) groups measured by a Corneometer on day 42. (C) Skin elasticity of CON (n = 5) and ABT-263-CGH (n = 5) groups evaluated by pinch test on day 42. (D, E) Representative H&E and Masson's trichrome staining of wound tissues collected from CON (n = 5) and ABT-263-CGH (n = 5) groups on day 42. (F) Quantification of follicle regeneration based on (D). (G) Quantification of collagen area based on (E). Values are presented as mean ± SEM. Statistical significance was determined using Student's t-test. *P < 0.05, **P < 0.01, ***P < 0.001.

Variables	DFU(n = 5)	Healthy(n = 5)	p value
Gender (M/F)	2/3	3/2	1.0000
Age (years)	63.4 ± 9.89	64.0 ± 8.57	0.9209
Blood glucose (mmol/L)*	17.08 ± 4.02	6.84 ± 2.07	0.0023
WBC count (10^9/L)*	10.66 ± 1.53	7.16 ± 1.06	0.0039
Neutrophil rate (%)*	70.92 ± 2.50	62.32 ± 4.73	0.0112
Lymphocyte rate (%)	17.49 ± 4.40	23.27 ± 10.78	0.3152
Ultrasensitive C-reactive protein (mg/L)*	69.88 ± 37.95	8.97 ± 3.62	0.0226
Hemoglobin (g/L)*	98.81 ± 8.17	125.42 ± 10.06	0.0020
RBC count (10^{12}/L)*	3.30 ± 0.28	4.38 ± 0.49	0.0046
Platelet count (10^9/L)*	355.62 ± 70.79	237.28 ± 33.33	0.0161
Aspartate aminotransferase(U/L)	35.16 ± 7.90	30.20 ± 10.08	0.4132
Alanine aminotransferase (U/L)	29.30 ± 6.33	26.16 ± 7.61	0.4991
Albumin (g/L)*	31.02 ± 2.45	37.50 ± 4.30	0.0247
Urea nitrogen (mmol/L)	7.76 ± 1.06	6.50 ± 0.78	0.0678
Serum creatinine (μmol/L)	108.16 ± 16.01	93.20 ± 16.76	0.1871
Serum uric acid (μmol/L)	388.80 ± 46.95	342.68 ± 36.93	0.1246
Low density lipoprotein (mmol/L)	3.99 ± 0.27	3.45 ± 0.45	0.0566
Total cholesterol (mmol/L)	5.89 ± 0.29	5.45 ± 0.68	0.2351
Triglyceride (mmol/L)	2.04 ± 0.62	1.48 ± 0.26	0.1194

Table S1. Clinical characteristics of diabetic foot ulcer (DFU) patients and healthy people . *$p < 0.05$

**Gene name**	**Primer sequence (5'-3')**	
Hsa-*GAPDH*	F: GGAGCGAGATCCCTCCAAAAT	R: GGCTGTTGTCATACTTCTCATGG
Hsa-*CDKN2A*	F: ATGGAGCCTTCGGCTGACT	R: GTAACTATTCGGTGCGTTGGG
Hsa-*CDKN1A*	F: CGATGGAACTTCGACTTTGTCA	R: GCACAAGGGTACAAGACAGTG
Hsa-*IL1A*	F: TGGTAGTAGCAACCAACGGGA	R:ACTTTGATTGAGGGCGTCATTC
Hsa-*IL1B*	F: ATGATGGCTTATTACAGTGGCAA	R: GTCGGAGATTCGTAGCTGGA
Hsa-*CXCL1*	F: AGCTTGCCTCAATCCTGCATCC	R: TCCTTCAGGAACAGCCACCAGT
Hsa-*MMP10*	F: TGCTCTGCCTATCCTCTGAGT	R: TCACATCCTTTTCGAGGTTGTAG
Mmu-*Gapdh*	F: AGGTCGGTGTGAACGGATTTG	R: TGTAGACCATGTAGTTGAGGTCA
Mmu-*Cdkn2a*	F: AACTCTTTCGGTCGTACCCC	R:GCGTGCTTGAGCTGAAGCTA
Mmu-*Cdkn1a*	F: CCTGGTGATGTCCGACCTG	R: CCATGAGCGCATCGCAATC
Mmu-*Il1a*	F: TCTATGATGCAAGCTATGGCTCA	R: CGGCTCTCCTTGAAGGTGA
Mmu-*Il1b*	F: GAAATGCCACCTTTTGACAGTG	R: TGGATGCTCTCATCAGGACAG
Mmu-*Cxcl1*	F: CTGGGATTCACCTCAAGAACATC	R: CAGGGTCAAGGCAAGCCTC
Mmu-*Mmp10*	F: GAGCCACTAGCCATCCTGG	R: CTGAGCAAGATCCATGCTTGG

Table S2. Sequences of Primers Required for qPCR. Hsa: Homo sapiens; Mmu: Mus Musculus.

k (h^{-1})	n	equation	r^2
0.55202	0.26602	y=0.26602x-0.59404	0.99083

Table S3. Release kinetics parameters of ABT-263-GE hydrogel
